# Genus-wide comparison of *Pseudovibrio* bacterial genomes reveal diverse adaptations to different marine invertebrate hosts

**DOI:** 10.1371/journal.pone.0194368

**Published:** 2018-05-18

**Authors:** Anoop Alex, Agostinho Antunes

**Affiliations:** 1 CIIMAR/CIMAR, Interdisciplinary Centre of Marine and Environmental Research, University of Porto, Porto, Portugal; 2 Department of Biology, Faculty of Sciences, University of Porto, Porto, Portugal; Zhejiang University, CHINA

## Abstract

Bacteria belonging to the genus *Pseudovibrio* have been frequently found in association with a wide variety of marine eukaryotic invertebrate hosts, indicative of their versatile and symbiotic lifestyle. A recent comparison of the sponge-associated *Pseudovibrio* genomes has shed light on the mechanisms influencing a successful symbiotic association with sponges. In contrast, the genomic architecture of *Pseudovibrio* bacteria associated with other marine hosts has received less attention. Here, we performed genus-wide comparative analyses of 18 *Pseudovibrio* isolated from sponges, coral, tunicates, flatworm, and seawater. The analyses revealed a certain degree of commonality among the majority of sponge- and coral-associated bacteria. Isolates from other marine invertebrate host, tunicates, exhibited a genetic repertoire for cold adaptation and specific metabolic abilities including mucin degradation in the Antarctic tunicate-associated bacterium *Pseudovibrio* sp. Tun.PHSC04_5.I4. Reductive genome evolution was simultaneously detected in the flatworm-associated bacteria and the sponge-associated bacterium *P*. *axinellae* AD2, through the loss of major secretion systems (type III/VI) and virulence/symbioses factors such as proteins involved in adhesion and attachment to the host. Our study also unraveled the presence of a CRISPR-Cas system in *P*. *stylochi* UST20140214-052 a flatworm-associated bacterium possibly suggesting the role of CRISPR-based adaptive immune system against the invading virus particles. Detection of mobile elements and genomic islands (GIs) in all bacterial members highlighted the role of horizontal gene transfer for the acquisition of novel genetic features, likely enhancing the bacterial ecological fitness. These findings are insightful to understand the role of genome diversity in *Pseudovibrio* as an evolutionary strategy to increase their colonizing success across a wide range of marine eukaryotic hosts.

## Introduction

Prokaryotes constitute a major portion of the Earth’s biota and around 60% of their global diversity is estimated to inhabit the oceanic environments. Bacterial species can have a free-living lifestyle in open waters, while some live in close association with a wide range of marine eukaryotes, in a host-dependent or symbiotic lifestyle [1]. The rapid advances in the sequencing of bacterial genomes have provided significant insights into the molecular mechanisms and evolutionary processes occurring during host-bacteria interaction and adaption [2]. Host-adapted bacteria exhibit diversity in genomic content enabling them to survive in different ecological niches. In the marine ecosystem, sponges (phylum *Porifera*) provide such niches by harboring remarkable diversity of microbes, which make them an important model system to investigate the molecular and evolutionary mechanisms of meatazoa-bacteria interactions [3]. Previous analyses of bacterial community in the sponge *Cymbastela concentrica* shed insight into processes such as the role of genomic factors- adhesion-related proteins, ankyrin repeat (ANK) proteins, and tetratricopeptide repeats domain-encoding proteins (TPR) responsible for bacteria-sponge interaction and abundance of transposable elements crucial for the evolution of symbiotic bacterial genomes [4–6]. Moreover, specific adaptations were identified in the hosts and the symbiotic minimalism among the sponge cyanobionts relatively to free-living counterparts [7].

Recently, the genus *Pseudovibrio* has become the focus of extensive research since its members dominate marine invertebrate-associated microbial communities and have showed the potential ability to produce novel compounds [8]. The genus *Pseudovibrio* consists of six type strains- *P*. *denitrifican*s [9] and *P*. *japonicus* [10] isolated from coastal seawater of Taiwan and Japan, respectively, *P*. *ascidiaceicola* isolated from the marine tunicate *Polycitor proliferus* [11], *P*. *axinellae* isolated from the marine sponge *Axinella dissimilis* [12], and *P*. *hongkongensis* [13] and *P*.*stylochi* [14] isolated from the marine flatworm *Stylochus* sp. Most of the strains were described as marine, heterotrophic, and facultative anaerobes capable of denitrification and fermentation. The members of *Pseudovibrio* were recurrently detected from various marine sources including tunicates [15,16], corals [17], algae [18] and a wide variety of globally distributed marine sponges [19–23]. The abundance of *Pseudovirbrio* in the larvae of sponge *M*. *laxissima* indicates the possible vertical transmission of *Pseudovibrio* within their hosts [20]. Hence, increasing evidence suggests that the *Pseudovibrio*-related bacteria might engage in symbiotic relationship with various marine invertebrate eukaryotic hosts.

Genome analyses of the genus *Pseudovibrio* representatives isolated from an enrichment culture of *Beggiatoa* sp. 35Flor, a filamentous sulfide-oxidizing bacterium sampled from a black band diseased scleractinian coral and several sponges across different geographical locations [24–26] suggested their genomic/metabolic versatility to thrive either as free-living or as host-associated. For example, comparative genomics analyses of *Pseudovibrio* isolated from a coral and sponges revealed the presence of gene clusters involved in symbiont-host interactions, horizontal gene transfer, genomic islands encoding toxin-immunity systems, several metabolic gene clusters in almost all bacterial members of the genus *Pseudovibrio*, and genome size reduction in a sponge-associated bacterium [24–26]. Nevertheless, there is a lack of knowledge on the genomic diversity and composition differences of other members of the genus *Pseudovibrio* with distinct lifestyles, such as associations with other marine invertebrates, namely tunicates [15] and flatworm, and the free-living members.

Here, we performed comparative genomic analyses of 18 genomes of the genus *Pseudovibrio* to uncover the gene repertoires involved in ecological diversity and adaptation. Our study revealed complex genomic patterns in the *Pseudovibrio* genus: (*i*) homogeneity among most of the sponge/coral-associated bacteria, (*ii*) flatworm-associated and some sponge/tunicate-associated members showing specialized adaptive requirements (e.g., genome reduction and distinct carbohydrate utilization). Noteworthy, the genes coding for symbioses factors (eukaryotic-like proteins) and effector molecules responsible for invasion/colonization were detected in all bacterial members of the genus *Pseudovibrio*. Our analyses also highlighted the presence of various defense systems, such as restriction modification (RM) and clustered regularly interspaced short palindromic repeats (CRISPRs). Furthermore, frequent detection of genomic islands (GIs) and mobilomes suggests the evolution of *Pseudovibrio* genomes through horizontal gene transfer. To our knowledge, this is the first comprehensive comparative genomics study among the genus *Pseudovibrio* revealing unique genomic signatures within bacterial species inhabiting different ecological niches.

## Materials and methods

### Genome sequence retrieval and annotation

A total of 18 bacterial genomes of the genus *Pseudovibrio* representing different ecological habitats were retrieved from the NCBI genomes FTP site (ftp://ftp.ncbi.nlm.nih.gov) (last accessed on 25^th^ April 2016) and the JGI genome portal (last accessed on 25^th^ Feb 2016). The isolation source of the bacteria and the assembly version of the related genomes are summarized in Table 1. All the contigs were re-ordered with MAUVE v.2.3.1 [27] using the complete genome of *Pseudovibrio* sp. FO-BEG1 as a reference sequence before proceeding with further analyses. In order to avoid the incongruence of different annotation systems, all the genomes were re-annotated using Prokka v1.10 [28], a standalone bacterial genome annotation pipeline. Contamination and completeness of the draft genomes of the genus *Pseudovibrio* were examined using CheckM v.1.0.4 [29] with the *Rhodobacteraceae* gene set as reference.

**Table 1:** Genome characteristic features of the bacterial species of the genus *Pseudovibrio* used for comparative genomic analyses.

Species	Habitat/host (isolation source)	Chromosome Size (bp)	G+C (%)	Genes (n)	CDS (n)	COGs (%)	Assembly version
*Pseudovibrio* sp. FO-BEG1	Scleractinian coral. ^(c)^	5475670	52.50	5042	4927	80.3	ASM143130v1
*Pseudovibrio* sp. JE062	*Mycale laxissima*^(s)^	5726521	52.40	5245	5099	81.3	ASM15623v1
*Pseudovibrio* sp. POLY-S9	*Polymastiapenicillus*^(s)^	6603616	51.26	6278	6171	69.8	ASM143130v1
*P*. *axinellae* AD2	*Axinelladissimilis*^(s)^	5126200	50.30	4710	4631	78.8	ASM162325v1
*Pseudovibrio* sp. AD13	*A*. *dissimilis*^(s)^	6001312	50.64	5571	5470	77.4	ASM162322v1
*Pseudovibrio* sp. AD14	*A*. *dissimilis*^(s)^	6201736	50.02	5765	5674	76.3	ASM162324v1
*Pseudovibrio* sp. AD26	*A*. *dissimilis*^(s)^	6181400	45.17	5787	5681	76.1	ASM162328v1
*Pseudovibrio* sp. AD37	*A*. *dissimilis*^(s)^	5875058	50.00	5547	5442	75.7	ASM162307v1
*Pseudovibrio* sp. AD46	*A*. *dissimilis*^(s)^	6124061	49.79	5602	5503	77.2	ASM162306v1
*Pseudovibrio* sp. AD5	*A*. *dissimilis*^(s)^	6061014	49.87	5591	5493	77.1	ASM162309v1
*Pseudovibrio* sp. W64	*A*. *dissimilis*^(s)^	5935921	50.13	5496	5405	78.2	ASM162314v1
*Pseudovibrio* sp. W74	*Haliclonasimulans*^(s)^	6190724	50.59	5774	5683	76.2	ASM162308v1
*Pseudovibrio* sp. WM33	*A*. *dissimilis*^(s)^	5745729	51.02	5425	5345	76.8	ASM162316v1
*P*. *ascidiaceicola*DSM-16392	*Polycitorproliferus*^(t)^	5845495	51.24	5411	5309	78.3	1096561*
*Pseudovibrio*sp. Tun.PHSC04_5.I4	*Synoicumadareanum*^(t)^	6549844	50.31	6215	6063	73.2	1075165*
*P*. *stylochi* UST20140214-052	*Stylochus* sp. ^(fw)^	3682052	47.02	3389	3300	82.2	ASM156205v1
*P*. *hongkongensis* UST20140214-015B	*Stylochus* sp. ^(fw)^	3746600	53.29	3567	3480	79.2	ASM156199v1
*P*. *denitrificans* JCM12308	Seawater^(fl)^	6054277	52.24	6228	6146	76.52	ASM131081v1

Single asterisk (*) represents accession numbers of genomes retrieved from the JGI portal. Letters in superscript represents different isolation sources- coral (c), sponges (s), tunicates (t), flatworm (fw), and free-living (fl). FO-BEG1 was isolated from *Beggiatoa* sp., bacterium initially sampled from a coral.

### Phylogeny of the genus *Pseudovibrio*

Maximum likelihood (ML) phylogenetic trees were constructed using the 16S rRNA and conserved marker genes, and a parsimony tree using the character matrix for presence/absence of genes. For the ‘whole genome tree’ analyses, amino acid sequences of 30 conserved marker genes were retrieved using AMPHORA2 [30]. The 16S rRNA and the marker genes were aligned using Clustal Omega [31] alignment algorithm implemented in SeaView v4.4.2 [32]. Poorly aligned positions of protein sequences were eliminated with Gblocks v0.19b using stringent parameters [33]. Final conserved blocks were concatenated to a super-alignment. ML trees were constructed with PhyML [34], resampled using 1000 bootstrap replicates. The choice of best-fit evolutionary models for 16S rRNA and protein sequences, TrN+I+G and LG+I+G+F, were calculated using MrAIC v1.4.4 [35] and ProtTest v3.4.2 [36], respectively. ML phylogenetic trees were rooted using closest relative *Pseudoxanthobacter soli* as an outgroup.

Genome-scale average nucleotide identity (ANI) among the genomes of all members of the genus *Pseudovibrio* was calculated using python module and scripts provided in pyani package [37] (https://github.com/widdowquinn/pyani) with the BLAST algorithm (ANIb) calculations. Circular maps are drawn using BLAST Ring Image Generator (BRIG v 0.95) [38].

### Clusters of orthologous groups of proteins

Clusters of orthologous groups (COGs) were annotated using a standalone RPS-BLAST v2.2.31 [39] against the pre-formatted (profile) RPS-BLAST database of CDD (conserved domains database) COG distribution constituting improved protein family annotations (ftp://ftp.ncbi.nih.gov/pub/mmdb/cdd/little_endian/) (accessed on November 2015) with an e-value cutoff of 0.001. The RPS-BLAST results were processed using *cdd2cog*.*pl* script v0.1 for further analyses (https://github.com/aleimba/bac-genomics-scripts). COG functional class abundance was represented by a heat map using heatmap.2 function in gplots package v 3.0.1 [40] implemented in R v3.2.4 [41]. Hierarchical clustering method based on euclidean distance was used to construct a dendrogram. Significant differences in proportions of COG categories were determined using Z-test. Categories with *p*-values of <0.05 were considered significantly different.

### Core- and pan-genome analyses

The core- and pan-genome sizes of the genus *Pseudovibrio* were estimated by clustering the coding sequences (CDS) using the bidirectional best-hit (BDBH), COGtriangles, and OrthoMCL clustering algorithms implemented in an open-source software package GET_HOMOLOGUES v2.0 [42] with 75% pairwise alignment coverage and E-value (expectation value for BLAST alignments) set at 1e-03. Due to the variation in orthologous cluster composition based on the clustering algorithm, we adopted a robust consensus approach, in which the gene clusters recognized by all three clustering algorithms were included in the subsequent analyses (using *compare_clusters*.*pl* script). Briefly, the core-genome was estimated using the consensus gene families defined by BDBH, COGtriangles, and OrthoMCL. Whereas, the pan-genome was estimated using the gene families defined by COGtriangles and OrthoMCL algorithms.

The theoretical size of the core- and pan-genomes was estimated with the OrthoMCL derived gene families by fitting Willenbrock exponential model [43] using *plot_pancore_matrix*.*pl* script.

### Scanning of carbohydrate-active enzymes, secretion systems, and ABC transporters

Protein sequences of the genus *Pseudovibrio* were screened for carbohydrate-active enzymes (CAzymes) by searching against annotated Hidden Markov Model (HMM) profiles of CAZyme proteins (dbCAN HMMs 5.0) in dbCAN database (accessed on December 2016) [44,45] using hmmscan. An e-value cutoff <10^−5^ was used to filter the results. The presence of type III and type VI secretion systems in all members of the genus *Pseudovibrio* was initially performed manually by inspecting the annotation files. BlastKOALA [46] tool was used for further classification and validation of both secretion systems using amino acid sequence of each genome as query against KEGG v2.1 [47]. Transporters were searched by initially predicting the transmembrane helices of the protein sequences using TMHMM server v2.0 [48]. Predicted proteomes were classified into transporters by performing BLASTP with an e-value cutoff of 10^−5^ against locally formatted Transporter Classification Database (TCDB) sequences (accessed on December 2016) [49]. The ATP-binding Cassette (ABC) transporters were parsed out for further analyses.

### Characterization of symbioses factors

To find the eukaryotic-like proteins (ELPs) containing motifs, such as tetratrico peptide repeats (TPRs), Ankyrin repeats (ANKs), Sel1 repeats, leucine-rich repeats (LRRs), fibronectin type III (fn3) domains, laminin G domains, and bacterial Ig-like domains, the protein sequences of the 18 *Pseudovibrio* genomes were scanned initially with the SMART (Simple Modular Architecture Research Tool) server [50]. TPRs/Sel1 motifs and LRRs were further predicted by a repeat sequence prediction tool, TPRpred v1.0 [51] and LRRfinder [52], respectively. The predicted genes encoding ELPs were back-searched against the Conserved Domain Database [53] by NCBI’s BLASTP service to avoid the false positive results.

InterProScan v5.24.63 [54] was used to scan for the genes containing YadA- (*Yersinia*adhesin A) (IPR005594), TadE-like (IPR012495) domains, and invasion associated locus B (*ialB*) protein (IPR010642). A representative set of genes coding for amyloid production and tight adherence (*tad*) locus of *Pseudovibrio* sp. FO-BEG1 was used to identify the homologous regions in other bacterial members using MultiGeneBlast v1.1.0 [55].

### Screening of CRISPR-Cas and restriction-modification systems

The genomes were searched for the presence of Clustered regularly interspaced short palindromic repeats (CRISPRs) with a webtool, CRISPRFinder [56] using default parameters. Questionable CRISPRs were omitted from the analyses. Cas (CRISPR-associated system) genes were identified by searching the annotation files considering the open reading frames (ORFs) including the term ‘cas’. Restriction-modification (RM) systems were estimated based on number of restriction enzymes detected in each genome. We searched the annotation files for the ORFs including the term ‘restriction’ in their annotation. A set of Pfam identifiers (PFAM family profile; S1 Table) curated in this study and described elsewhere [57] was used to identify the RM systems. Identified restriction enzymes were verified by BLAST similarity search against the restriction enzyme database REBASE [58]. We considered a RM system ‘complete’ and restriction enzyme (REase) and methyltransferase (MTase) as ‘solitary’ as mentioned previously [59]. Briefly, complete RM system was indicated by the syntenic presence of REase and MTase, whereas absence of either REase or MTase at a distance less than 10 genes were considered as ‘solitary’ REase and MTase.

### Mobile elements and subcellular localization analysis

IslandViewer 3 [60] was applied to infer the genomic islands (GIs) using an integrated prediction method based on SIGI-HMM [61], IslandPick [62], and IslandPath-DIMOB [63]. Before uploading to IslandViewer server, draft genomes were re-ordered using the reference sequence *Pseudovibrio* sp. FO-BEG1 and concatenated to a single sequence using union utility in the EMBOSS package [64]. We considered the GIs, when genomic regions are predicted by all three methods implemented in IslandViewer. PHASTER [65] was used to predict the phage elements with default parameters. In order to detect the transposons outside the prophage regions, individual annotation files were screened using the keyword ‘transpos’. Functional characterization and subcellular localization of the genes in GIs were performed based on COG classification as mentioned above and PSORTb v3.0.2 [66], respectively. Comparison of the proportion of functional genes encoded in the genomes of *Pseudovibrio* and their genomic islands was performed using *t*-test. Categories with *p*-values of <0.05 were considered significantly different.

## Results and discussion

### Genome summary of the genus *Pseudovibrio*

The characteristic genomic features of the genus *Pseudovibrio* (n = 18) are summarized in Table 1. The *Pseudovibrio* sp. isolated from different hosts namely coral (FO-BEG1 was isolated from an enrichment culture of *Beggiatoa* sp. 35Flor sampled from a coral—thus, FO-BEG1 will be mentioned as coral-associated strain throughout the text), sponge-associated (JE062, POLY-S9, AD2, AD13, AD14, AD26, AD37, AD46, AD5, W64, W74, WM33), tunicate-associated (DSM-16392, Tun.PHSC04_5.I4), flatworm-associated (UST20140214-052, UST20140214-015B), and free-living (JCM12308), showed varying genome sizes. The larger genome size of 6.6 Mbp was observed for the *Pseudovibrio* sp. POLY-S9 isolated from intertidal marine sponge *Polymastia penicillus* [24] and the smallest genome of 3.6 Mbp for the *P*. *stylochi* UST20140214-052 isolated from a marine flatworm. Estimations of the completeness and contamination of the genomes of the genus *Pseudovibrio* indicate that almost all the bacterial genomes are near complete (≥ 90%) and exhibiting low levels of putative contamination (≤ 5%) (S1 Fig).

### Lack of host-specific phylogenetic clustering

**Fig 1:**
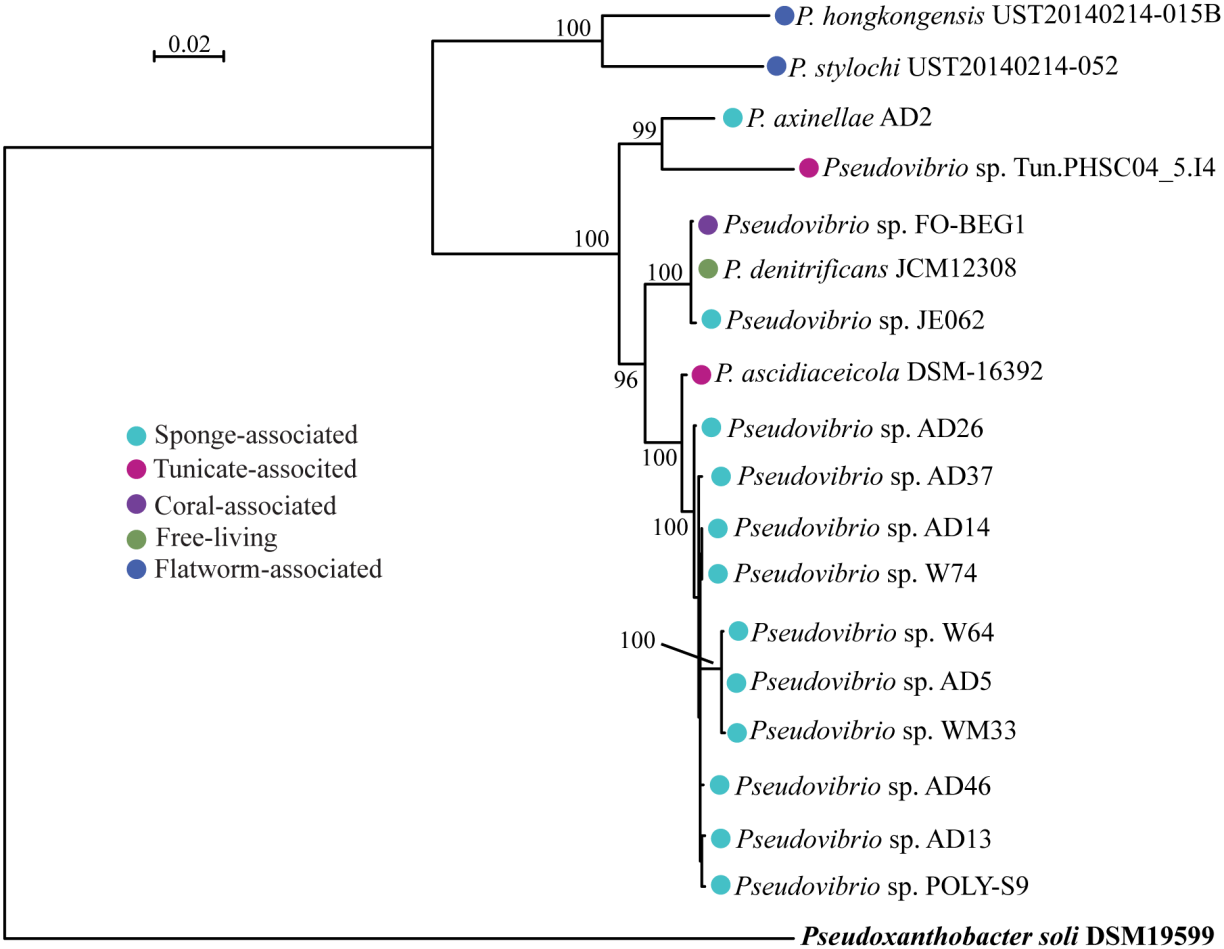
Evolutionary relationship of the genus *Pseudovibrio* inferred by maximum likelihood (ML) analyses. ML tree was constructed using a concatenated super-alignment of 30 marker genes (*dnaG*, *frr*, *infC*, *nusA*, *pgk*, *pyrG*, *rplA*, *rplB*, *rplC*, *rplD*, *rplE*, *rplF*, *rplK*, *rplL*, *rplM*, *rplN*, *rplP*, *rplS*, *rplT*, *rpmA*, *rpoB*, *rpsB*, *rpsC*, *rpsE*, *rpsI*, *rpsJ*, *rpsK*, *rpsS*, *smpB*, *and tsf*) representing 7.7 kb. The *rpsM* gene was not considered in the analyses since it is missing in the genome of POLY-S9. Different colored shapes indicate the isolation sources. Bootstrap support values are shown at each node of the phylogenetic tree. The tree is rooted using an outgroup shown in bold.

Previous studies involving the phylogenetic reconstruction using 16S rRNA and core-genome indicated that the members of the genus *Pseudovibrio* failed to cluster based on the isolation sources (e.g. host-associated) [25,26]. Our extended phylogenetic analyses using 30 marker genes (~7.7 Kbp) (‘whole genome tree’) of the 18 *Pseudovibrio* genomes isolated from the different marine invertebrates further affirm the lack of host-specific clustering (Fig 1). For instance, the free-living bacterium JCM12308 was grouped with the host-associated bacteria JE062 and FO-BEG1 isolated from a sponge and *Beggiatoa* sampled from a diseased coral. Whereas, the nine Irish sponge-associated bacteria grouped with another sponge-associated bacterium POLY-S9 isolated from the Atlantic coast and a tunicate-associated bacterium *P*. *ascidiaceicola*. Lack of clustering was further supported by the 16S rRNA phylogeny (S2 Fig) and a parsimony tree built based on the presence/absence of genes (S3 Fig). However, it is noteworthy that the two bacteria- *P*. *stylochi* and *P*. *hongkongensis* isolated from the same flatworm specimen (*Stylochus* sp.) grouped together and were considerably divergent from all the other members, suggesting likely their independent evolution from other host-associated bacterial members of the genus *Pseudovibrio*. The genetic relatedness of these bacteria was further confirmed by ANI (S4 Fig). Current phylogenetic branching might also indicate two distinct patterns of evolution among the genus *Pseudovibrio*: (*i*) the host-switching ability of the *Pseudovibrio* members enabling them to colonize wide marine invertebrate hosts (e.g., sponges, tunicates, and corals), and (*ii*) the specialized association with a marine flatworm through genome streamlining. Overall these results highlight the versatile nature of the genus *Pseudovibrio* ranging from host-associated to free-living, and their ability to adapt and survive in different habitats.

### Core- and pan-genome of the genus *Pseudovibrio*

The robust estimation of core- and pan-genome analyses using a consensus orthologous clustering approach (see Materials and methods) of 94,822 protein CDS from the 18 genomes of the genus *Pseudovibrio* identified a total of 13,226 clusters defining the pan-genome (S5A and S5B Fig). Out of these, 11.1% were recognized in all the bacterial members analyzed here. Statistical estimation of the theoretical size of the core- and pan-genomes using Willenbrock exponential model based on orthoMCL clustering indicated a decrease in the core- and an increase in the pan-genome size. It is also clear that the pan-genome fitting curve does not reach a plateau (S5C and S5D Fig), suggesting that the pan-genome of *Pseudovibrio* is open.

**Fig 2:**
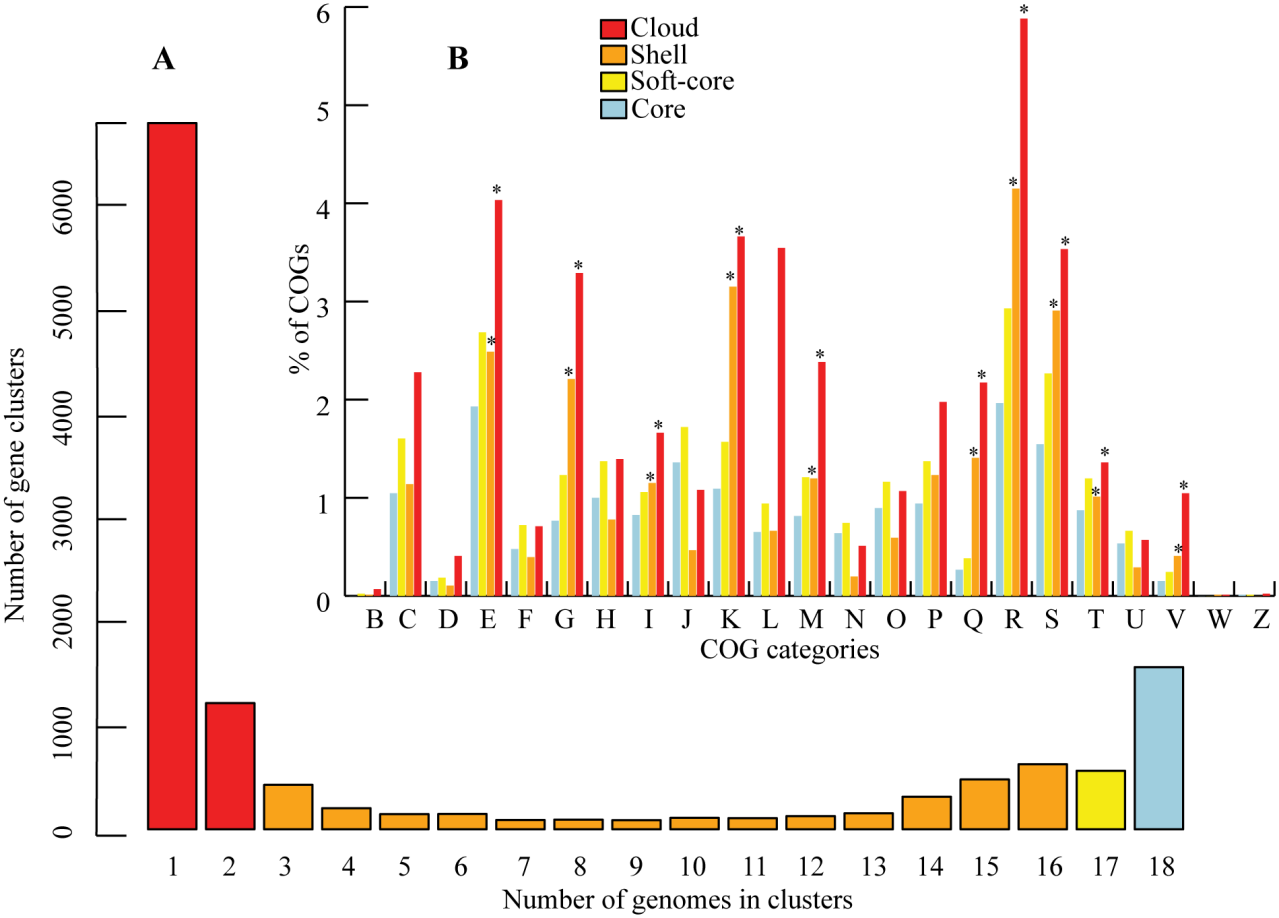
Structure of the pan-genome. (A) Bar plot showing the frequencies of orthologous clusters. (B) Bar plot of percentage of COGs assigned to each cluster- (‘cloud’, ‘shell’, ‘soft-core’, and ‘core’). Individual COG categories were determined based on the number of COGs assigned to genes in each clusters. The asterisks denote the significant difference (p<0.05) of COG categories observed among the different clusters.

The less conserved compartment of the pan-genome structure (‘soft-core’, ‘shell’, and ‘cloud’) was further computed (Fig 2A). We considered the ‘soft-core’ genome as representing the conserved genes across 95% of the genomes since it allows analyzing the draft genomes even if some genes could be missing. The ‘shell’ cluster includes the genes detected in the majority of the genomes (moderately common genes), while the ‘cloud’ cluster constitute the genes observed in the minority of the genomes [67]. Thus, the less conserved ‘cloud’ and ‘shell’ clusters representing a subset of the flexible genome could be used to infer the evolutionary history and lifestyle-specific adaptation of an organism [68]. Functional annotation of the genes in the universally distributed core-genome (encompassing information of 18 genomes) and the less conserved flexible-genome was performed by RPS-BLAST (see Materials and methods). The COGs were assigned to 94%, 92.4%, 63.5%, and 41% of the core, soft-core, shell, and cloud genes, respectively. An overrepresentation of COG categories (Z-test, *p*<0.005, Fig 2B, S2 Table) was detected in the flexible-genome relative to the core-genome.

A previous study comparing *Pseudovibrio* strains isolated from sponges reported some degree of homogeneity in the number of orthologous genes [26]. Estimation of strain-specific genes using a subset of the flexible-genome (‘cloud’ clusters) indicated a similar tendency in terms of genes shared amongst most of the sponge/coral-associated bacteria (S6 Fig). Higher fraction of the COG category responsible for transcription (K) and replication, recombination, and repair (L) in the Antarctic tunicate-associated bacterium Tun.PHSC04_5.I4 suggests better adaptability to the local environment due to the fact that COG category K contains many transcriptional regulators. The higher number of 16S rRNA operons (n = 9) in the genome of Tun.PHSC04_5.I4 further validates the increased translation efficiency in the cold habitat [69]. Supporting this statement, we detected homologues of multiple copies of cold-shock proteins (Csp) in the genome of Tun.PHSC04_5.I4. During cold-shock, bacteria experience decrease in membrane fluidity, reduced translation and transcription, inefficient protein folding, and inability of ribosomes to function properly (as reviewed in [70]). Bacteria respond to rapid temperature drop through the synthesis of cold-shock proteins, which enable them to adapt and overcome these challenges [71]. Proteins homologous to CspA are widely detected in prokaryotes living in extreme conditions and in *Escherichia coli* environmental stress has been associated with a larger number of proteins of the CspA family originated by gene duplications [72]. Multiple copies (4 copies) of the genes coding for cold-shock protein A (*cspA*) in Tun.PHSC04_5.I4 when compared to other *Pseudovibrio* genomes (2 copies of *cspA*) suggest the ability of *Pseudovibrio* sp. Tun.PHSC04_5.I4 to thrive and adapt in close-association with the Antarctic tunicate host. Other characteristic features of AD2, UST20140214-052, and UST20140214-015B are discussed in the following sections.

### Diversity of CAZymes

**Fig 3:**
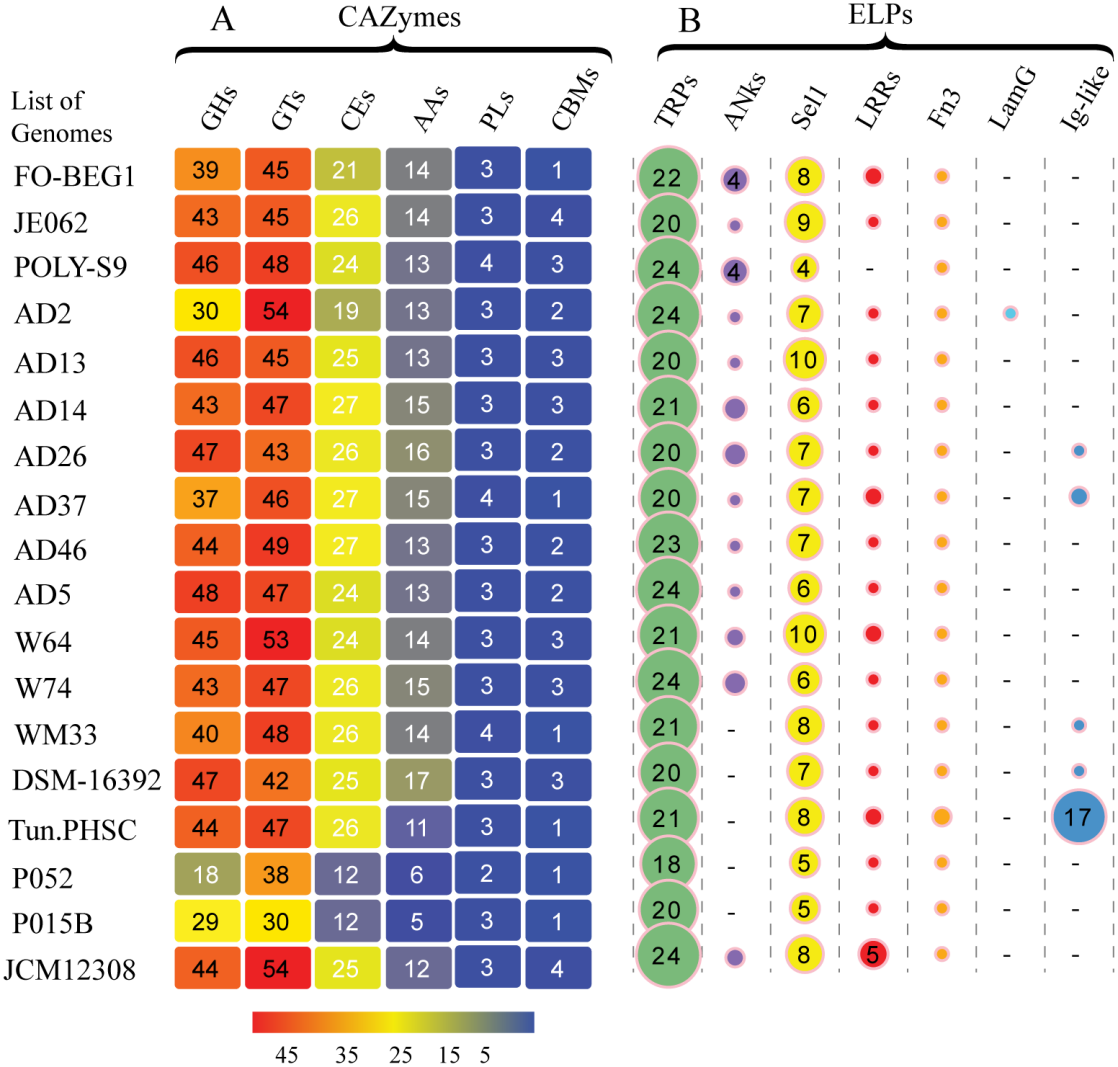
Predicted carbohydrate-active enzyme (CAZyme) and eukaryotic-like proteins (ELPs) in the genus *Pseudovibrio*. (A) Heat map representation of CAZYme repertoire. Numbers of each enzyme detected in the genome are shown as overrepresented (red) and underrepresented (blue). (B) The bubble sizes are proportional to the number of each ELPs detected. The absence of ELPs is represented by a hyphen (-). List of the genomes are abbreviated using the strain information (see Table 1).

Carbohydrate metabolism is a crucial step enabling the survival of microbes living in diverse habitats. Genome-wide screening for carbohydrate-active enzymes (CAZymes) in the 18 *Pseudovibrio* bacteria retrieved various functional classes: glycoside hydrolases (GHs), glycosyltransferases (GTs), polysaccharide lyases (PLs), carbohydrate esterases (CEs), carbohydrate-binding modules (CBMs), and auxiliary activities (AAs), suggesting the ability of the analyzed members of the genus *Pseudovibrio* to metabolize different carbohydrates in diverse habitats (Fig 3A). The presence of the glycoside hydrolase family 109 possessing an α-N-acetylgalactosaminidase activity as a shared and most abundant GH family suggests the ability of the host-associated *Pseudovibrio* (sponge/tunicate) to degrade the glycoproteins/glycoconjugates in the sponge and tunicate cell wall matrix. A similar trend was previously reported among the sponge symbiont *Poribacteria* [73]. Apart from the presence of shared GH families, detection of unique GHs might indicate specific adaptation of certain *Pseudovibrio* species to utilize and degrade carbohydrates. For instance, detection of GH116 and GH129 (α-*N*-acetylgalactosaminidase), which has been implicated in alternative mucin degradation pathway [74] in the genome of a tunicate-associated *Pseudovibrio* sp. Tun.PHSC04_5.I4, suggest the ability of the bacterium to utilize the mucous secretion of the tunicate. It is known that tunicates filter the food particles from the seawater using a complex mucous secretion [75] and a previous study detected expression of vertebrate gel-forming mucins in the epithelial cells covering the body of the tunicate *Oikopleuradioica* [76]. However, GH116 and GH129 were not detected in *P*. *ascidiaceicola*, another tunicate-associated bacterium analyzed here, which might be due to the host-specific changes in carbohydrate utilization by *Pseudovibrio*.

### Abundance of eukaryotic-like proteins and other symbioses factors

The protein-coding genes containing the eukaryotic-like motifs/eukaryotic-like proteins (ELPs) such as ANKs (CL0465), TPRs (CL0020), LRRs (CL0022), Sel1-like repeats (PF08238; CL0020), Fn3-like (PF14310; CL0159), laminin-G -3 (PF13385; CL0004), and bacterial Ig-like domains (PF02369; CL0159) involved in protein-protein interactions for a range of cellular processes were detected in almost all members of the genus *Pseudovibrio* (Fig 3B, S3 Table). Among them, three ELPs—TPRs, Sel1, and Fn3 were detected in all the 18 *Pseudovibrio* sp. analyzed here. An abundance of ELPs- TPRs, ANKs, Sel1, LRRs, Fn3, LamG, and Ig-like have been reported in many sponge-associated microbes suggesting the role of the genes containing repeat protein domains for establishing successful association with the hosts [4–6,73,77]. The role of ELPs (ANKs) in sponge-bacteria interaction was further validated experimentally elsewhere [78]. A comprehensive study reported that rather than lifestyle or pathogenicity, the phylogenetic history is a determinant factor of TPR abundance in bacteria [79]; whereas, ANK abundance is determined by lifestyle rather than phylogenetic history [80]. However, we detected different types of ELPs in varying proportions in both host-associated and free-living members of the genus *Pseudovibrio*. For instance, abundance of LRRs and TPRs were detected in a free-living member. Previous studies show that LRRs are essential for the virulence of pathogenic bacteria *Yersinia pestis* [81] and trigger the host-cell invasion by the pathogen *Listeria monocytogenes* [82]. Whereas, TPR class of proteins found in facultative and symbiotic bacteria are essential for evading the host immune response by modulating the eukaryotic protein-protein interactions. The presence of some eukaryotic-like proteins in non-host associated members might suggest an ancient origin of these ELPs [83]. Detection of more ELPs, especially LRRs and TPRs, may suggest the ability of this strain to infect different range of marine invertebrate hosts. Another plausible explanation is that the members of the genus *Pseudovibrio* likely adapt to various environmental niches by procuring distinct ELPs through HGT (some ELPs were detected within the GIs), which warrants further investigation.

In addition to ELPs, we detected genes probably involved in cell surface adhesion and host invasion (S3 Table). For instance, YadA- and TadE-like domains responsible for surface adherence and binding to the host tissue were detected in *Pseudovibrio* strains isolated from different marine sources (YadA was not detected in AD2, UST20140214-052, and UST20140214-015B). Metaproteogenomic analysis reported the presence of the adhesion YadA among the microbial communities associated with the sponge *Cymbastela concentrica* [77]. *Yersinia* adhesin A (YadA) is a cell adhesion factor and virulence determinant among many pathogenic bacteria, which mediates host cell adherence by binding to extracellular matrix (ECM) molecules, such as laminin, collagen, and fibronectin [84], and phagocytosis resistance [85]. Detection of the genes responsible for surface attachment is noteworthy due to the fact that ECM of sponges/tunicates is rich in peptidoglycans, fibronectin, laminin, and collagen [86–88]. Furthermore, homologues of invasion associated locus B (*ialB*) genes were detected in all the members (host-associated and free-living) of the genus *Pseudovibrio* (S3 Table). IalB is known as a major virulence factor in *Bartonella bacilliformis* responsible for adherence and invasion of human erythrocyte [89].

The genomes of *Pseudovibrio* (except in AD2, UST20140214-052, and UST20140214-015B) were encoded with the homologues of gene clusters responsible for bacterial amyloid (curli) production (S7A Fig). Curli fibrils are proteinaceous extracellular matrix frequently detected in both pathogenic and non-pathogenic bacteria [90,91]. These virulence factors are involved in bacterial attachment to cell surface, cell aggregation, and biofilm formation, and mediate the host-cell invasion [92]. Consistent with a previous report [25], we detected the homologues of *csgG* and *csgF* genes responsible for amyloid production and the genes coding for curlin associated repeat within the close proximity. In addition, we found the *tad* (tight adherence) locus in all the bacterial members analyzed here (S7B Fig). The *tad* genes are required for the assembly of adhesive Flp (fimbrial low-molecular-weight protein) pili, and are essential for the biofilm formation, colonization, and pathogenesis in many bacterial members [93]. Conclusively, the identified ELPs domains/gene clusters involved in adhesion, invasion, and colonization, further support the ability of *Pseudovibrio* to attach and survive in various marine invertebrate hosts.

### T3SS/T6SS and distribution of effector molecules

**Fig 4:**
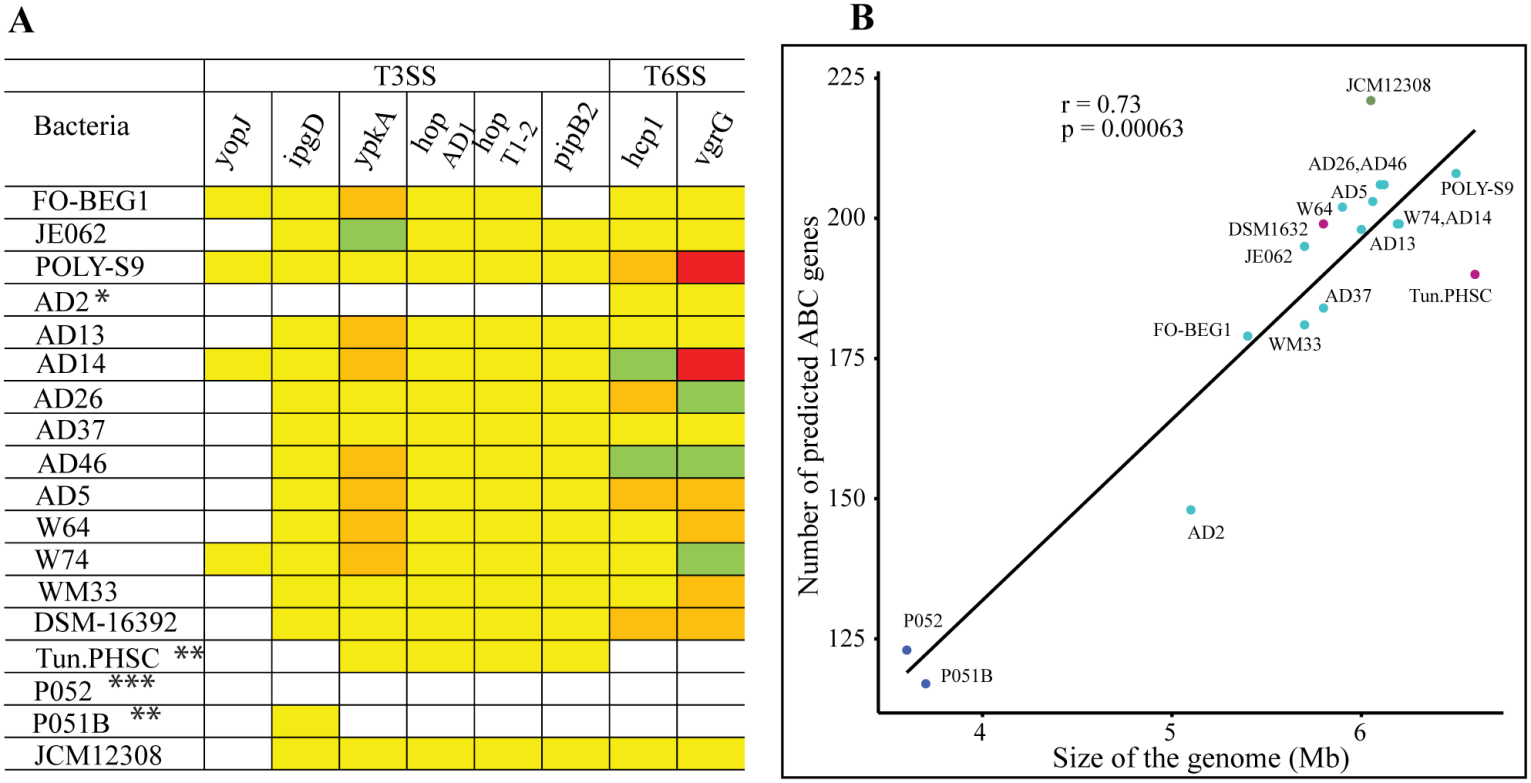
Predicted effector molecules and ABC transporter genes of the genus *Pseudovibrio*. (A) The frequency of T3 and T6 secretion system effector genes is indicated: 0 (white), 1–2 (yellow), 3 (orange), 4–5 (light green), and 6–7 (red). Single and double asterisks represent the strains devoid of T3SS and T6SS respectively. Whereas, three asterisk represent the strain without both secretion systems. (B) Comparison of number of predicted ABC transporter genes and the genome size of the members of the genus *Pseudovibrio*. The analysis was performed using spearman correlation test. Spearman rho (r) and *p*-values are given inside the plot. List of the genomes are abbreviated using the strain information (see Table 1).

Bacteria living in association with eukaryotic hosts utilize a multitude of molecular mechanisms to initiate a successful colonization. One of such strategies is the secretion of proteins/toxins- effector molecules and its transport across the membrane through various secretion systems (SSs) [94]. Such two SSs- T3SSs and T6SSs are important virulence factors believed to confer fitness to Gram-negative bacteria adapted to pathogenic and symbiotic lifestyles [95,96]. Previous genome analyses of *Pseudovibrio* sp. isolated from different sources revealed the variation in the genetic architecture of the secretion systems (SSs) and its possible role in initiating the host-bacteria interactions [24–26]. Our comparative analysis incorporating new genomes isolated from different niches (tunicates, flatworm, and free-living) revealed the lack of SSs in some members of the genus *Pseudovibrio* and non-uniform distribution of common effector molecules (Fig 4A). For instance, the genome of UST20140214-052 (flatworm-associated) lacked the gene cluster coding for both type III and type VI secretion systems. Whereas, type VI secretion system was absent in the genomes of both UST20140214-015B (flatworm-associated) and Tun.PHSC04_5.I4 (tunicate-associated). The lack of T3SSs has been reported in the extensively studied plant pathogen *Pseudomonas syringae*, presumably by allowing the better growth on the plants on which any of its effectors elicited the effector-triggered immunity (ETI) [97,98] and widen the plant host range [99]. A recent study reported the absence of T3SSs, suggesting the possible genome reduction event in *Pseudovibrio* sp. AD2 isolatedfrom a marine sponge [26]. We speculate that a similar genome reduction event might have occurred among both flatworm-associated *Pseudovibrio* strains. Considering the widespread distribution of the genus *Pseudovibrio*, we hypothesize that the absence of T3SSs and T6SSs may have resulted as an adaptation to reduce the fitness costs of host-specific virulence in an ecological niche, or the loss of SSs may have occurred during the course of a lifestyle adaptation.

These SSs apparatus deliver the effector proteins across the bacteria and into the cytosol of the host cells to gain control by modulating a variety of host cell functions, namely disruption of host cytoskeleton, immune and defense response. In addition to the previously reported genes encoding homologues of type III effector proteins YopJ (*Yersinia* outer protein), IpgD (inositol phosphate phosphatase), and YpkA (*Yersinia* protein kinase), other effector molecules- HopAD1, HopT1-2 (Hrp outer protein), and PipB2 (Pathogenicity island-encoded protein) were also detected (Fig 4A). Most common type VI secretion system effector molecules, Hcp1 (Hemolysin-coregulated protein) and RhsGE-associated Vgr family subset (COG3501)-vgrG-like protein (Valine-glycine repeat), were also detected in multiple copies. It is noteworthy that fewer T3SS/T6SS effector molecules were detected in the free-living bacterium *P*. *denitrificans* JCM12308 when compared to the host-associated bacteria. The possible roles of SSs and secreted proteins (*yopJ*, *ipgD*, *ypkA*) were reported previously among many sponge-associated bacteria [24,25]. The role of other T3Es- HopAD1 and HopT1-2 in suppressing the plant immunity and a complex interplay between various T3Es was extensively studied among the plant pathogen *Pseudomonas syringae* [100,101], whereas PipB2 act as a virulence factor involved in mediating the recruitment of kinesin-1 on the *Salmonella*-containing vacuole (SCV) for the maintenance of intracellular pathogenic lifestyle [102]. Conclusively, it further affirms that both host-associated and free-living bacteria of the genus *Pseudovibrio* deploy various and large repertoire of effector proteins to enable a series of events for the successful colonization and subversion of the host immune system. However, further experimental validation is required to understand the interplay among effector molecules in marine invertebrates.

### ABC transporters among the genus *Pseudovibrio*

Transporters belonging to the members of the ATP-binding Cassette (ABC) superfamily facilitate the translocation of a wide variety of solutes in and out of the cells. Bacterial ABC transporters play a major role in virulence through the uptake of nutrients, secretion of toxins and antimicrobial agents, and quorum sensing [103]. We investigated the relationship between the genome size and the numbers of encoded ABC transporter systems in the *Pseudovibrio* species. In accordance with a previous report [104], a correlation between the genome size and the total number of predicted ABC transporter genes was observed (*p* = 0.01) (Fig 4B). However, we found a higher number of ABC transporter encoding genes in *P*. *denitrificans* JCM12308 bacterium with a genome size relatively smaller when compared to a few other genomes here studied. It indicates that the bacterium with free-living/extracellular lifestyle might encounter variable environmental conditions, which in turn may demand an increased ABC system [105]. Most of the sponge-associated bacteria (except in *P*. *axinellae* AD2) studied here showed a tendency to encode more or less the same number of ABC genes further affirming the uniformity among these genomes and the ability of the bacterial members for extensive import of metabolites.

### The genus *Pseudovibrio* rely more on RM systems than in CRISPR-Cas systems

Evolutionary arms race between prokaryotes and phages resulted in the emergence of self defense mechanisms, namely CRISPR-Cas and RM systems, which oppose the invading foreign genetic materials. CRISPR-Cas is an abundant form of heritable adaptive immunity systems acting against invading specific viruses and often the immunological memory last through the lifetime of an individual [106]. By contrast, RM systems provide prokaryotes a non-specific protection through innate immunity [107]. In total, we identified 21 ‘complete’ RM systems and several ‘solitary’ REases and MTases in the genus *Pseudovibrio* (S4 Table). Type I RM systems were the most abundant (~66%) followed by Type II RM systems (~28%). The detection of RM systems in 11 genomes suggests the dependence of an alternative defense mechanism in the genus *Pseudovibrio*. A recent study reported less prevalence and complete absence of CRISPR-Cas systems across major bacterial lineages and symbionts. However, they detected other defense systems like integrated viral genes, RM, and abortive infection [108].

**Fig 5:**
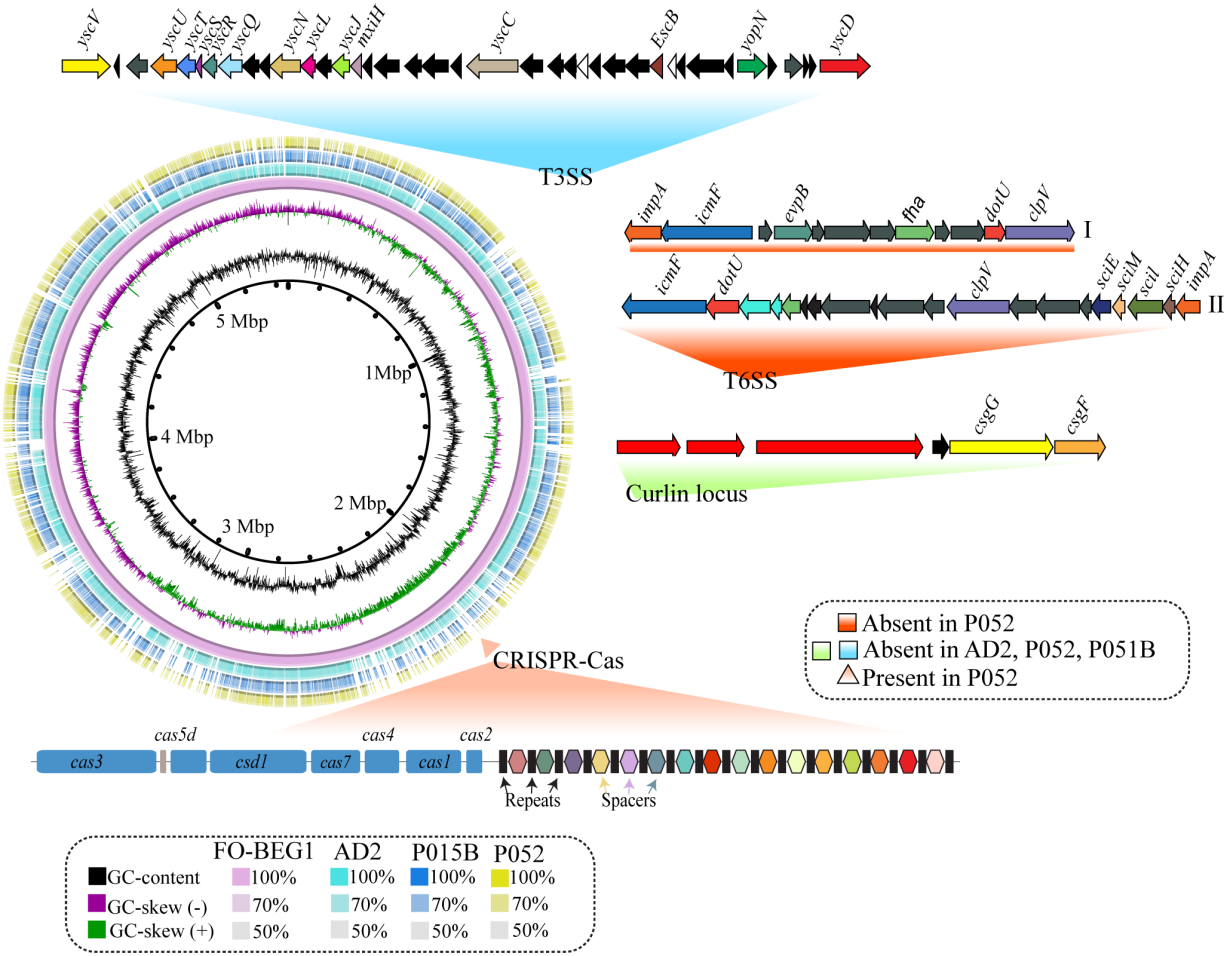
Genome-wide BLAST comparison of proposed minimal genomes of *P*. *axinellae* AD2, *P*. *stylochi* UST20140214-052, and *P*. *hongkongensis* UST20140214-015B with *Pseudovibrio* sp. FO-BEG1. Innermost circle represent the raw skeletal structure. Second, third, and fourth circles represent GC-content, GC-skew, and all predicted ORFs (open reading frames) of the reference genome FO-BEG1. Fifth, sixth, and seventh circles denotes similarity of predicted ORFs shared among AD2, UST20140214-052, and UST20140214-015B. Major gene clusters absent and detected in the genomes of AD2, UST20140214-052, and UST20140214-015B are indicated outside the circle. Absent gene clusters from top to clockwise direction- type 3 secretion systems (T3SSs), cluster I and cluster II of type 6 secretion system (T6SSs), and curlin locus. The only gene cluster, CRISPR-Cas detected in UST20140214-052 was represented at the bottom of the ring. Seven CRISPR associated genes (CAS)—*cas3*, *cas5d*, *csd1*, *cas7*, *cas4*, *cas1*, and *cas2* are shown in blue color. CRISPR locus detected downstream of CAS operon are represented by repeats (black) and spacers (colored diamond shapes).

Metagenomic studies identified an overrepresentation of CRISPR repeats and the genes encoding CRISPR-associated proteins, as well as RM systems in sponge-associated microbes when compared with surrounding seawater [4,6,109,110]. CRISPR loci and associated genes were only detected in the genomes of the sponge symbiont “*Candidatus* Synechococcus spongiarum”, when compared to their free-living relatives [7]. The above mentioned studies concluded that the sponge-associated microbes tend to harbor more genes responsible for self-defense due to a high filtration rate or frequent exposure to phage particles. Conversely, in our study none of the members of the genus *Pseudovibrio* associated with sponges had CRISPR-Cas systems, which was only detected in the genome of a flatworm-associated bacterium *P*. *stylochi* UST20140214-052 (Fig 5). The CRISPR locus consisted of an array of repeats of 32 bp long and 16 unique spacer sequences of 32–36 bp long. Seven *cas* genes were found immediately downstream of the CRISPR array (Fig 5). The conserved arrangement of *cas* genes suggests that the detected CRISPR-Cas in the genome of the *P*. *stylochi*UST20140214-052 belong to type I-C systems according to a newly proposed classification scheme [111]. We also detected the CRISPR locus (but not *cas* genes) in the genomes of AD37 and DSM-16392.

Although both *P*. *stylochi* UST20140214-052 and *P*. *hongkongensis* UST20140214-015B are isolated from the same flatworm specimen, thus experiencing similar environmental pressure, it is intriguing that only the former possessed the CRISPR-Cas system. Wepropose that (*i*) the presence of the CRISPR loci in the absence of *cas* genes indicate an early phase of CRISPR-Cas locus erased in some members of the genus *Pseudovibrio* and (*ii*) the possible role of RM systems in the genus *Pseudovibrio* as a first line of defense. Furthermore, current comparative genome analyses suggest that sponge-associated bacteria, particularly the members of the genus *Pseudovibrio* perhaps do not rely entirely on the CRISPR-Cas self-defense systems. An exhaustive search for other defense-related proteins and its role in the bacterial members of the genus *Pseudovibrio* would be insightful.

### Genomic islands and ecological fitness of the genus *Pseudovibrio*

**Fig 6:**
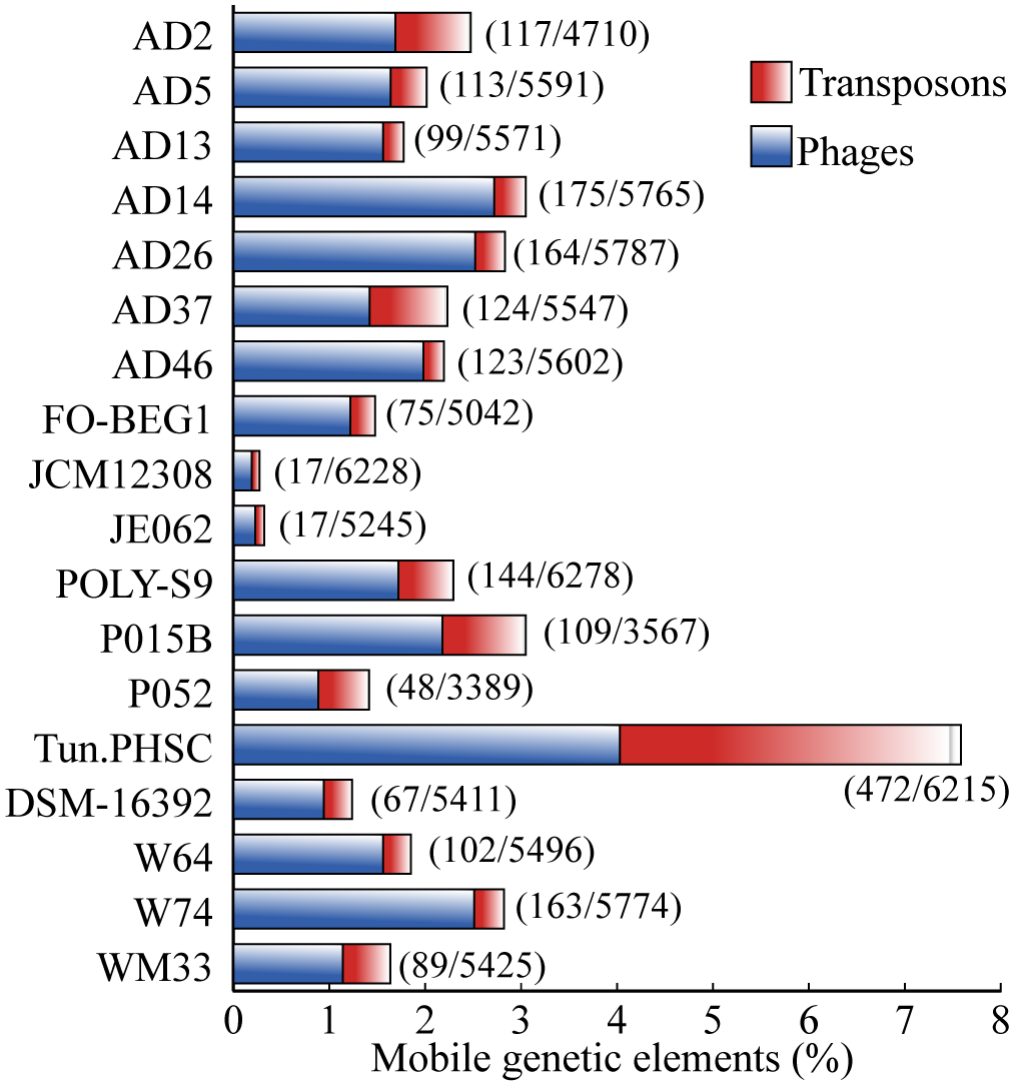
Mobilome composition in the genomes of the genus *Pseudovibrio*. The percentage of mobile elements (transposons and phages) encoding genes is represented as a staked bar graph. Numbers in parenthesis represent the predicted MGEs and genes in each genome.

Genomic islands (GIs) are another important contributing factor for the bacterial genome evolution and adaptation ranging from drug resistance to pathogenesis/symbioses [112]. GIs constitute clusters of genes in the genomes that reveal the evidence of horizontal gene transfer (HGT) [113]. Detection of mobilomes (phage particles and transposons) (Fig 6) and GIs in all the members (S8A Fig) suggests that HGT is frequent in the genus *Pseudovibrio* irrespective of the lifestyle, supporting the statement that HGT occur commonly in bacteria that have access to a horizontal gene pool [114]. It has been reported that subsets of genes with specific functions tend to encode within the GI regions [115]. The complexity hypothesis states that informational genes (transcription and translation) are less likely to be horizontally transferred than operational (housekeeping) genes [116]. To assess the complexity hypothesis among the members of the genus *Pseudovibrio*, we performed the comparison of the proportion of functional categories between the genomes and the GIs in order to infer the nature of functions being manifested within the GIs as mentioned elsewhere [117]. Consistent with the complexity hypothesis and a previous report [115], specific subsets of operational genes, COG ‘D’ (cell cycle control, cell division, chromosome partitioning), ‘L’ (replication, recombination and repair), ‘M’ (cell wall/membrane/envelope biogenesis), ‘Q’ (secondary metabolite biosynthesis, transport and catabolism), and ‘V’ (defense mechanisms), were significantly (t-test, *p*<0.005) overrepresented in GIs (S8B Fig, S5 Table). It is noteworthy that we detected a deviation from the complexity hypothesis due to the overrepresentation of informational genes- COG ‘K’ (transcription) in the GIs of the genus *Pseudovibrio*, a trend detected in the bacterial GIs analyzed elsewhere [115]. Some of the relevant COG classes detected are discussed below.

Glycosyltransferases involved in the cell wall biogenesis (COG0463 and COG0438) was detected in the GIs of *Pseudovibrio* bacterial members, suggesting the possible acquisition of the genes encoding the cell wall biosynthesis and the modification of cell surface [118]. Prevalence of genes encoding for a family of proteins, Repeats-in-toxin (RTX) (COG2931) was also detected in the GIs of the *Pseudovibrio* genomes. The majority of these toxin proteins identified belong to serraylisin-like metalloprotease and hemolysin. Toxin-like proteins act as synergistic virulence factors mediating mechanisms involved in facilitating the colonization of eukaryotic hosts [119]. In the defense mechanisms category, more genes related to COG1136 (ABC-type antimicrobial peptide transport system, ATPase component), COG0577 (ABC-type antimicrobial peptide transport system, permease component), and COG1566 (Multidrug resistance efflux pump) were detected. These putative ABC transporters predicted to be involved in the cell defense were reported in the genomes of the psychrophilic archaeon, *Methanococcoides burtonii*, and a marine bacterium *Pseudoalteromonas tunicata* for their successful adaptation to cold environment [120], and surface-associated lifestyle [121]. Abundance of genes belonging to certain COG classes indicate the possible procurement of operational genes involved in the biosynthesis of the cell wall, virulence proteins, transporters, and export of potential defense compounds. This suggests that the genus *Pseudovibrio* might experience complex lifestyle like host-switching /free-living, and under these conditions, HGT of the genes responsible for gene expression regulation could enable the rapid adaptation to changing environments [122].

Furthermore, we compared the proportion of the genes encoded for subcellular localization in the genomes and GIs, which revealed a significant higher percentage of genes belonging to the categories “Unknown” and “Extracellular” (t-test, *p*<0.05) in the GIs of the *Pseudovibrio* (S8C Fig, S6 Table). A higher percentage of genes in GIs with unknown subcellular localization might be due to the absence of orthologous matches in the database, which are novel/recently acquired and uncharacterized [123]. A greater proportion of genes belonging to extracellular categories could be explained by the fact that GIs of the *Pseudovibrio* encoded several sets of virulence-related genes, namely RTX proteins. It further suggests the possible role of GIs in implanting virulence-related genes (namely beta-lactamase HcpC, leukotoxin, hemolysin)—crucial for the host colonization and to evade the host immune system. The association of virulence genes with GIs have been previously reported [124], and it appears that GIs provide a certain fitness advantage [125] to the bacterial members of the genus *Pseudovibrio*. However, more genomes of *Pseudovibrio* from different habitats would be required to better assess the role of GI encoded genes in lifestyle-specific adaptation.

## Conclusions

Here we performed comparative genomics analyses to characterize the unique genomic features of *Pseudovibrio* find in association with marine eukaryotic hosts like sponges, coral, flatworm, and tunicates, and as a free-living bacterium. Pan- and core-genome analyses indicated that the genus *Pseudovibrio* has an open-pan genome. We propose a possible genome reduction event among the three *Pseudovibrio* sp. (AD2, UST20140214-052, and UST20140214-015B) due to the absence of certain genetic features, namely (*i*) secretion systems and virulence/effector proteins, (*ii*) symbioses factors responsible for attachment to the eukaryotic hosts, and (*iii*) reduced number of ABC transporters. However, such hypothesis should be interpreted with caution. Despite the absence of above mentioned genetic architectures, the comparative genomic analyses shows that all the three *Pseudovibrio* with reduced genome sizes encoded the major essential metabolic pathways crucial for its survival in nutrient-poor environment. Our study further suggests that genomic islands and mobilomes might be responsible for the genome plasticity and horizontal gene transfer among the *Pseudovibrio* species, which possibly favor the colonization of invertebrate hosts in various habitats. Such genome-level findings provide insight into the evolutionary adaptation and the genomic versatility of the genus *Pseudovibrio*. Moreover, our comparative genomic study of *Pseudovibrio* species is likely to represent only a subset of the genomic diversity representing this abundant host-associated group of bacteria, but it should provide the basis for future extensive comparative analyses of larger numbers of bacterial members isolated from different habitats.

## Supporting information

S1 Fig Visual representation of the completeness and contamination within each genome of the genus *Pseudovibrio*. A total of 19 *Pseudovibrio* sp. were checked for completeness and contamination here before short listing 18 genomes for further comparative genomic analyses. Single copy markers identified are represented by green bars. Contamination within the genome is represented in a scale of 2 to 5 by color (yellow to red) and grey represents missing markers. The strain heterogeneity is indicated in a scale of 2 to 5 by color ranging from light-blue to dark-blue.
(TIF)

S2 Fig Maximum-likelihood phylogenetic tree inferred using 16S rRNA genes of *Pseudovibrio* species. Color codes represent the isolation sources. Bootstrap support values are shown at each node. The tree is rooted using an outgroup shown in bold.
(TIF)

S3 Fig Parsimony tree based on the presence-absence pan-genome matrix. The phylogeny was reconstructed using Fitch parsimony algorithm implemented in GET_HOMOLOGUES (see Materials and methods). Strains are color coded according to the isolation source.
(PNG)

S4 Fig Heat map of ANIb percentage identity for 18 isolates of *Pseudovibrio* spp. used in current study. Species identifiers using their corresponding codes are given as row and column labels. The color scale from shade of white (low) to green (high) represents the similarity of the bacteria.
(TIF)

S5 Fig Core- and pan-genome analyses of the genus *Pseudovibrio*. Venn diagram representing the consensus (A) core- and (B) pan-genome clusters computed using the respective clustering algorithms: COG, OMCL, and BDBH. Statistical estimation of (C) the core- and (D) pan-genome sizes of the genus *Pseudovibrio*. The curves are fitted proposed by Willenbrock exponential model based on the orthoMCL clustering.
(TIF)

S6 Fig Predicted strain-specific genes. (A) Bar graph showing total number of strain-specific genes estimated from the ‘cloud’ cluster and (B) Heat map representation of COG functional assignment are shown.
(TIF)

S7 Fig Schematic representation of curli assembly and *tad* loci in the genomes of *Pseudovibrio*. Arrows indicate the relative location and direction of transcription of ORFs. Predicted ORFs of the similar function are represented by same color. ‘X’ denotes the absence of gene cluster in respective species. The ORFs are not drawn to scale.
(TIF)

S8 Fig Genomic islands (GIs) and functional annotation of GI genes. (A) Number of GIs detected in the genus *Pseudovibrio*, (B) COG functional classification of the genes within the GIs, and (C) predicted subcellular localization genes within the GIs. Stacked bar graph represents the fraction of genes within the GI. Different color codes represent each functional category.
(TIF)

S1 Table List of Pfam identifiers used to query RM systems in the genomes of the genus *Pseudovibrio*. (DOCX)

S2 Table Prorportion of COG categories detected in the core and flexible (shell and cloud) genomes. (XLSX)

S3 Table List of predicted eukarytic-like proteins (ELPs) in the genomes of the genus *Pseudovibrio*. (XLSX)

S4 Table List of predicted restriction-modification systems in the 18 genomes of the genus *Pseudovibrio*. (DOCX)

S5 Table Comparison of proportion of functional genes encoded in the *Pseudovibrio* genomes and their genomic islands. *P*-values are based on t-test.
(DOCX)

S6 Table Comparison of proportion of genes encoded for subcellular localization in the *Pseudovibrio* genomes and their genomic islands. *P*-values are basedon t-test.
(DOCX)
