# Supplementary figures and images for: Genus-wide comparison of Pseudovibrio bacterial genomes reveal diverse adaptations to different marine invertebrate hosts
Source: PLoS One. 2018 May 18;13(5):e0194368. doi: 10.1371/journal.pone.0194368 (PMC5959193; doi:10.1371/journal.pone.0194368)

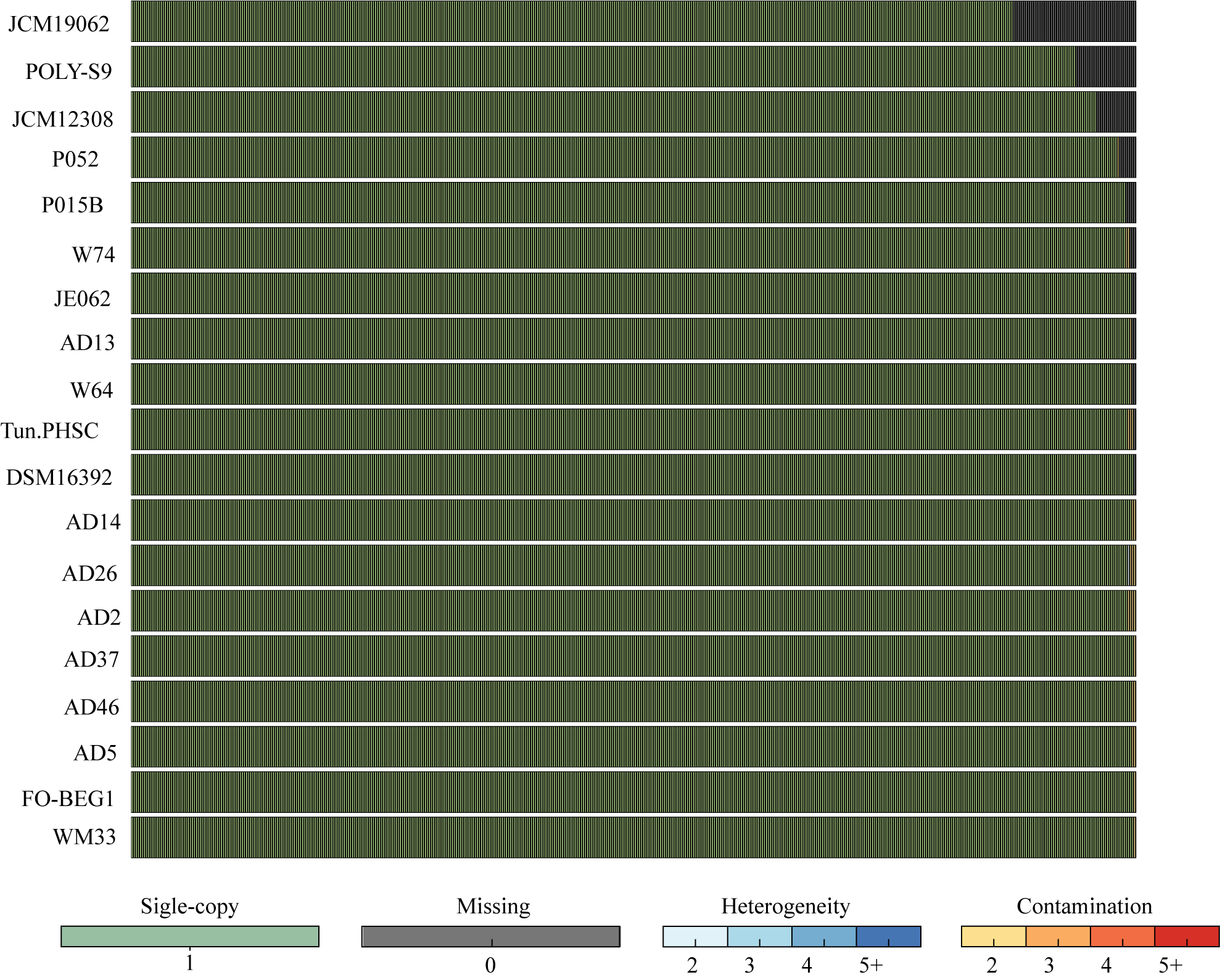

Supplement: S1 Fig — A total of 19 Pseudovibrio sp. were checked for completeness and contamination here before short listing 18 genomes for further comparative genomic analyses. Single copy markers identified are represented by green bars. Contamination within the genome is represented in a scale of 2 to 5 by color (yellow to red) and grey represents missing markers. The strain heterogeneity is indicated in a scale of 2 to 5 by color ranging from light-blue to dark-blue. (TIF) [file pone.0194368.s001.tif]

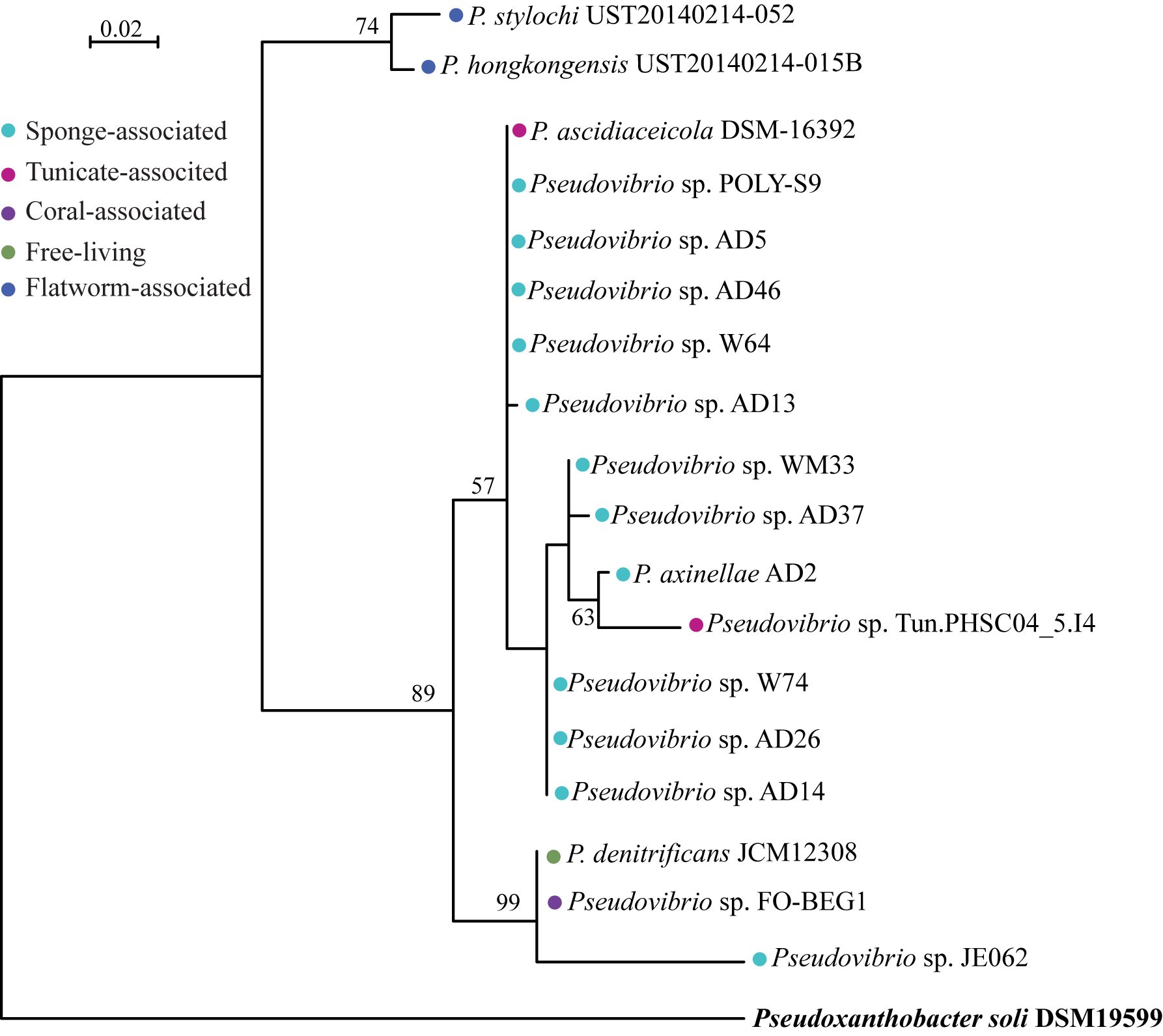

Supplement: S2 Fig — Color codes represent the isolation sources. Bootstrap support values are shown at each node. The tree is rooted using an outgroup shown in bold. (TIF) [file pone.0194368.s002.tif]

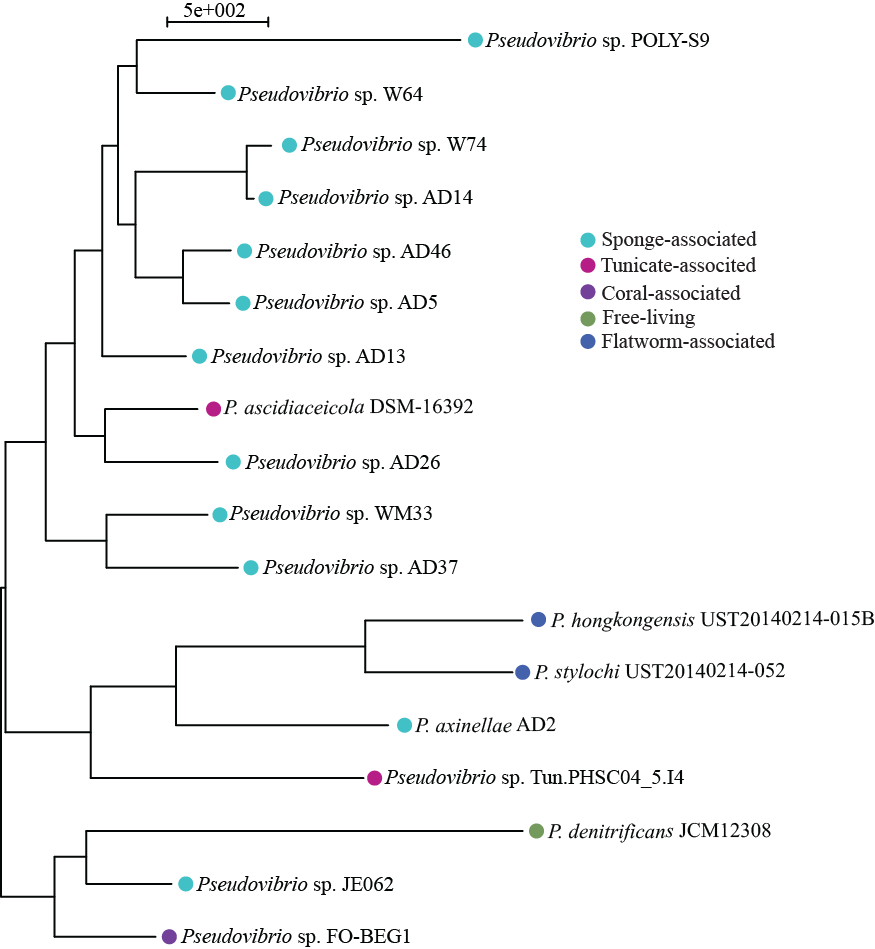

Supplement: S3 Fig — The phylogeny was reconstructed using Fitch parsimony algorithm implemented in GET_HOMOLOGUES (see Materials and methods). Strains are color coded according to the isolation source. (PNG) [file pone.0194368.s003.png]

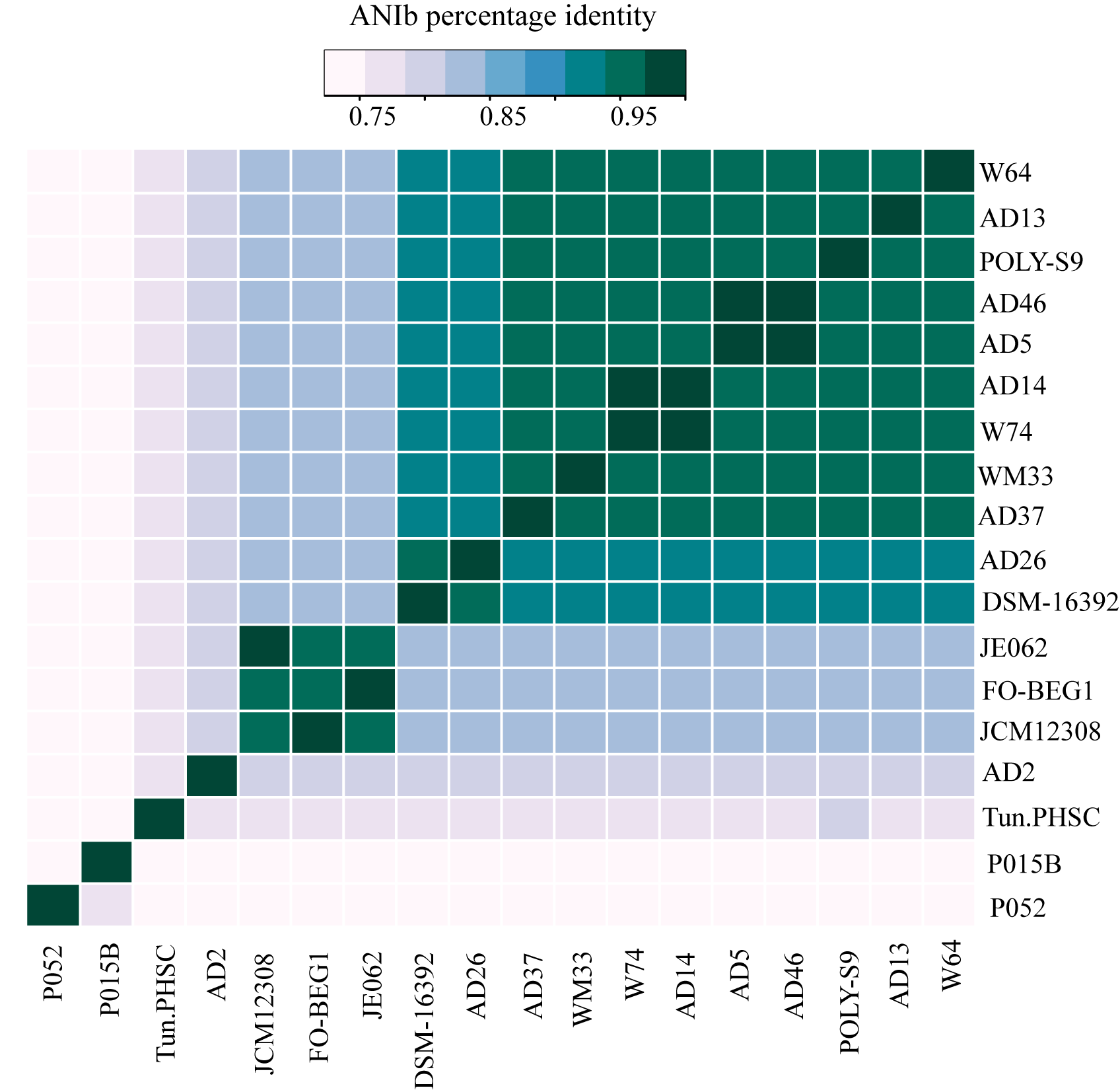

Supplement: S4 Fig — Species identifiers using their corresponding codes are given as row and column labels. The color scale from shade of white (low) to green (high) represents the similarity of the bacteria. (TIF) [file pone.0194368.s004.tif]

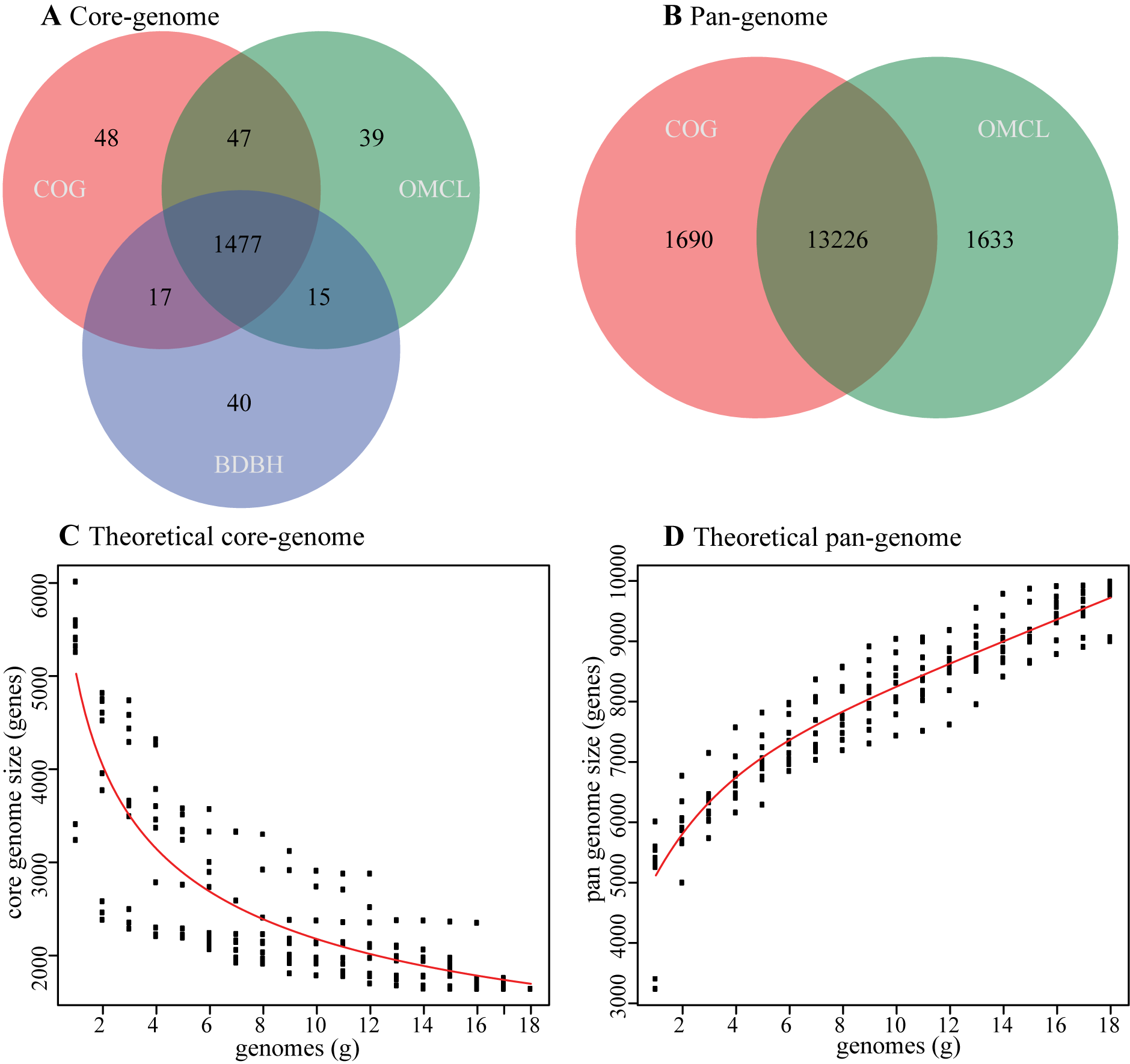

Supplement: S5 Fig — Venn diagram representing the consensus (A) core- and (B) pan-genome clusters computed using the respective clustering algorithms: COG, OMCL, and BDBH. Statistical estimation of (C) the core- and (D) pan-genome sizes of the genus Pseudovibrio. The curves are fitted proposed by Willenbrock exponential model based on the orthoMCL clustering. (TIF) [file pone.0194368.s005.tif]

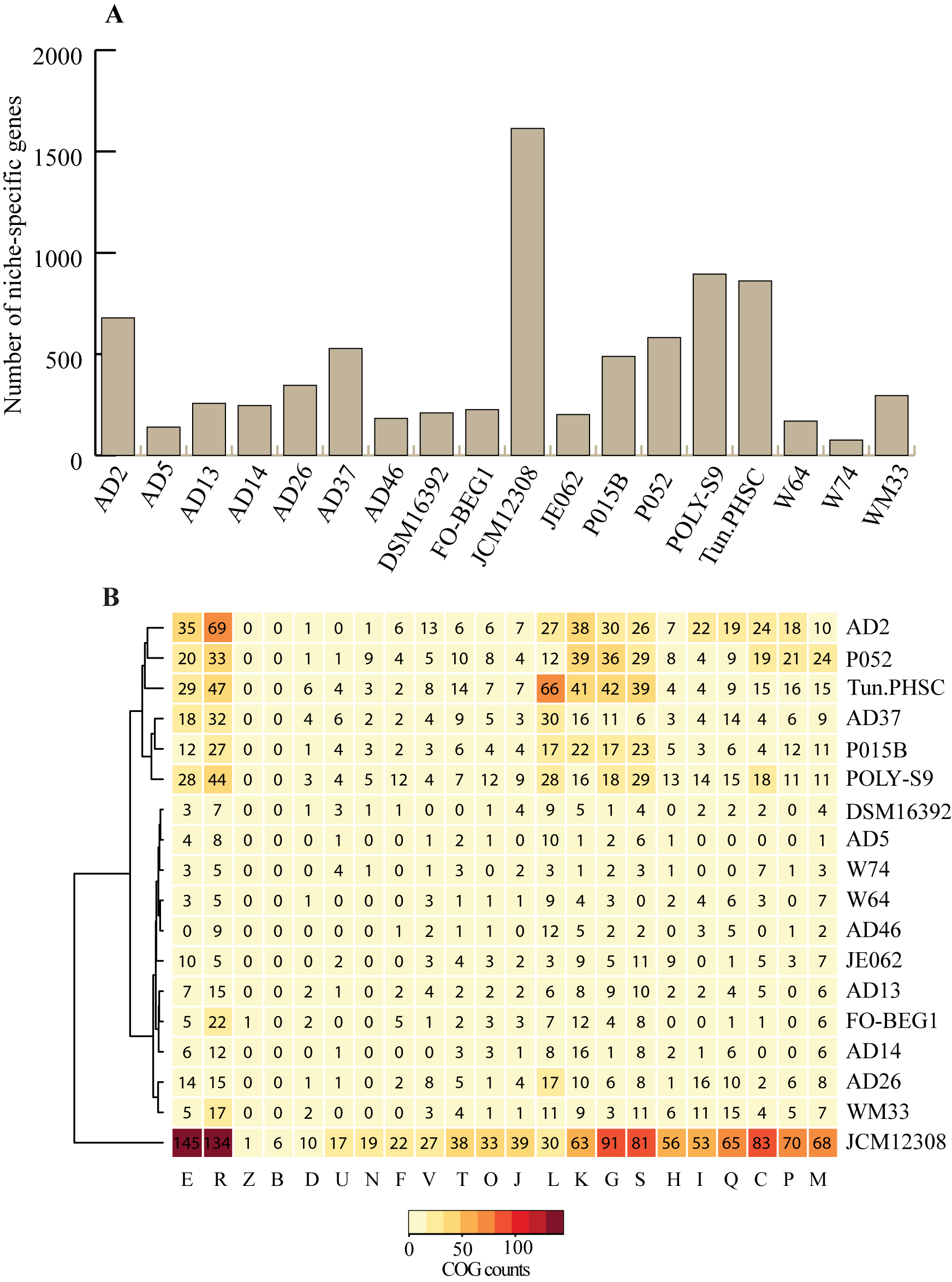

Supplement: S6 Fig — (A) Bar graph showing total number of strain-specific genes estimated from the ‘cloud’ cluster and (B) Heat map representation of COG functional assignment are shown. (TIF) [file pone.0194368.s006.tif]

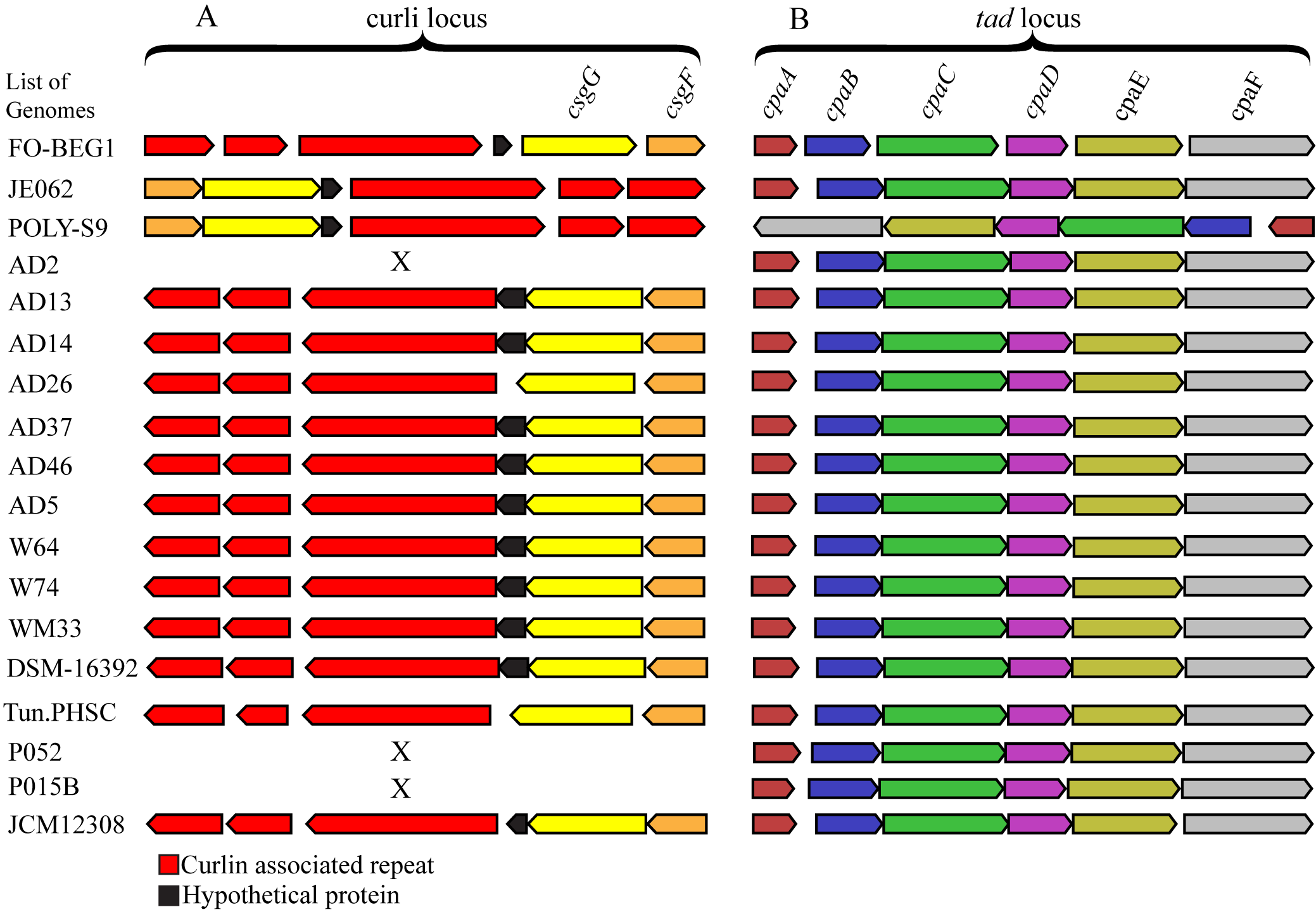

Supplement: S7 Fig — Arrows indicate the relative location and direction of transcription of ORFs. Predicted ORFs of the similar function are represented by same color. ‘X’ denotes the absence of gene cluster in respective species. The ORFs are not drawn to scale. (TIF) [file pone.0194368.s007.tif]

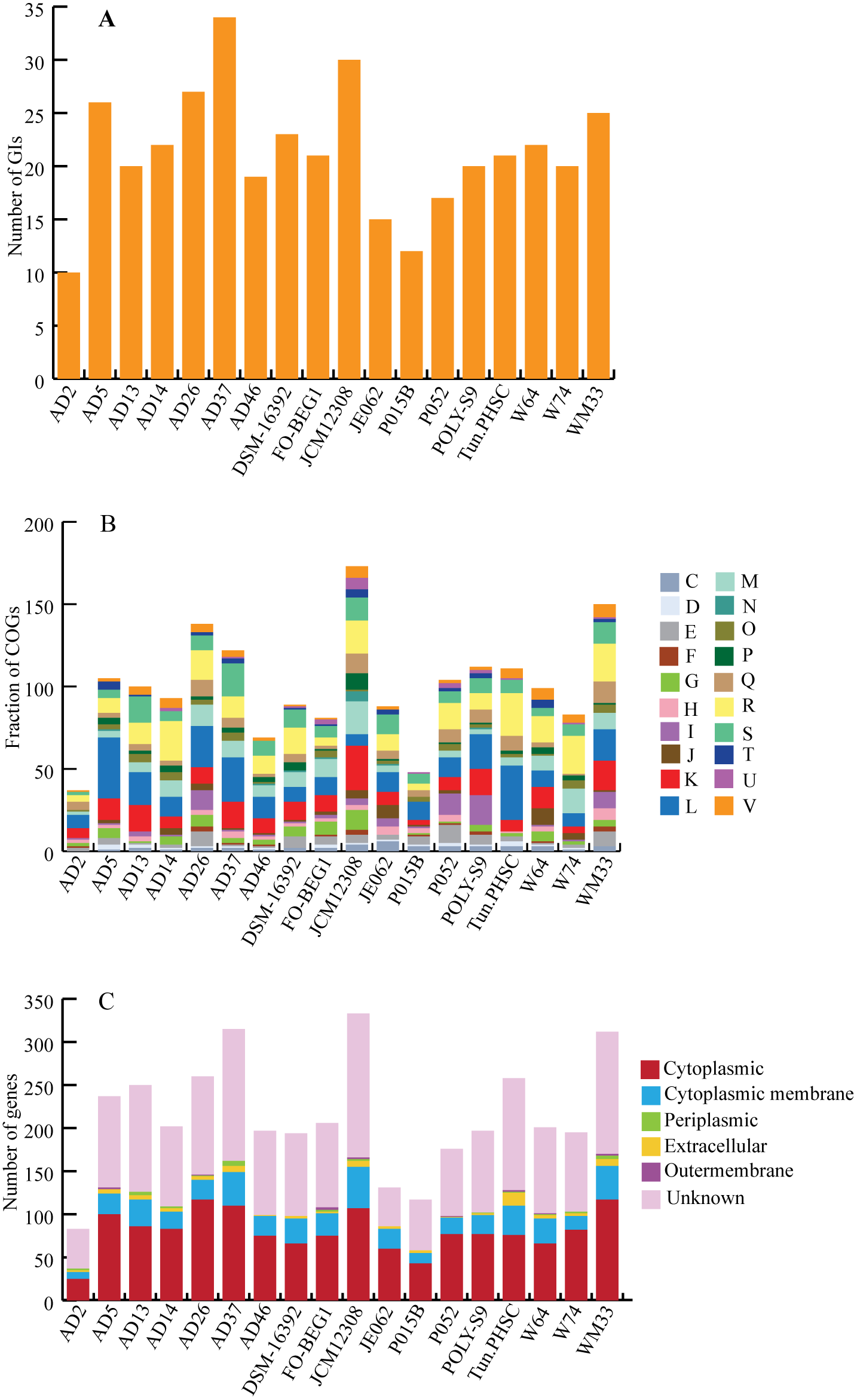

Supplement: S8 Fig — (A) Number of GIs detected in the genus Pseudovibrio, (B) COG functional classification of the genes within the GIs, and (C) predicted subcellular localization genes within the GIs. Stacked bar graph represents the fraction of genes within the GI. Different color codes represent each functional category. (TIF) [file pone.0194368.s008.tif]
