# Supplementary material for: Genus-wide comparison of Pseudovibrio bacterial genomes reveal diverse adaptations to different marine invertebrate hosts
Source: PLoS One. 2018 May 18;13(5):e0194368. doi: 10.1371/journal.pone.0194368 (PMC5959193; doi:10.1371/journal.pone.0194368)
Supplement: S1 Table — (DOCX) [file pone.0194368.s009.docx]

**Table S1**. List of Pfam identifiers used to query RM systems in the genomes of the genus *Pseudovibrio*

| **Pfam Identifier** | **Name of restriction and enzymes** |
| --- | --- |
| PF10592 | AIPR |
| PF03230 | Antirrestrict |
| PF07275 | ArdA |
| PF02923 | BamH1 |
| PF11564 | BpuJI_N |
| PF07832 | Bse6341 |
| PF06616 | BsuBI_PstI_RE |
| PF12106 | Colicin_C |
| PF04556 | DpnII |
| PF06044 | DRP |
| PF08011 | DUF1703 |
| PF08819 | DUF1802 |
| PF12957 | DUF3846 |
| PF13020 | DUF3883 |
| PF13643 | DUF4145 |
| PF04411 | DUF524 |
| PF07669 | Eco571 |
| PF08463 | EcoEI_R_C |
| PF12008 | EcoE124_c |
| PF02963 | EcoRI |
| PF09019 | EcoRIILC |
| PF09217 | EcoRIILN |
| PF09195 | Endonuc-BglII |
| PF09194 | EndonucLBsobI |
| PF09233 | EndonucLEcoRV |
| PF09254 | EndonucLFokI_C |
| PF09226 | EndonucLHincII |
| PF09208 | EndonucLMspI |
| PF09225 | EndonucLPvuII |
| PF02980 | FokI_C |
| PF02981 | FokI_N |
| PF08797 | HIRAN |
| PF12161 | HsdM_N |
| PF04313 | HSDR_N |
| PF13588 | HSDR_N_2 |
| PF09509 | Hypoth_Ymh |
| PF14354 | Lar_restr_alle |
| PF10117 | McrBC |
| PF01420 | Methylase_S |
| PF04471 | Mrr_cat |
| PF13156 | Mrr_cat_2 |
| PF14338 | Mrr_N |
| PF02384 | N6_Mtase |
| PF09126 | NaeI |
| PF09015 | NgoMIV_restric |
| PF12183 | NotI |
| PF08684 | ocr |
| PF11463 | RLHINP1I |
| PF04002 | RadC |
| PF11058 | RaI |
| PF04851 | ResIII |
| PF11407 | RestrictionMunI |
| PF11487 | RestrictionSfiI |
| PF09545 | RE_AccI |
| PF09665 | RE_Alw26IDE |
| PF09491 | RE_AlwI |
| PF09499 | RE_ApaLI |
| PF09549 | RE_Bpu10I |
| PF09504 | RE_Bsp6I |
| PF09552 | RE_BstXI |
| PF09516 | RE_CfrBI |
| PF09517 | RE_Eco29kI |
| PF09553 | RE_Eco47II |
| PF09554 | RE_HaeII |
| PF09556 | RE_HaeIII |
| PF09518 | RE_HindIII |
| PF09519 | RE_HindVP |
| PF09561 | RE_HpaII |
| PF09563 | RE_LlaJI |
| PF09562 | RE_LlaMI |
| PF09567 | RE_MamI |
| PF09568 | RE_MjaI |
| PF09564 | RE_NgoBV |
| PF09565 | RE_NgoFVII |
| PF09521 | RE_NgoPII |
| PF09522 | RE_R_Pab1 |
| PF09566 | RE_SacI |
| PF09569 | RE_ScaI |
| PF09570 | RE_SinI |
| PF09573 | RE_TaqI |
| PF09572 | RE_XamI |
| PF09571 | RE_XcyI |
| PF13707 | RloB |
| PF06300 | Tsp45I |
| PF12564 | TypeIII_RM_meth |
| PF05685 | Uma2 |
| PF04555 | XhoI |
| PF09520 | RE_MjaII |
| PF01555 | N6_N4_Mtase |
| PF02086 | MethyltransfD12 |
| PF00145 | DNA_methylase |
| PF07669 | Eco57I |
