# Supplementary material for: Genus-wide comparison of Pseudovibrio bacterial genomes reveal diverse adaptations to different marine invertebrate hosts
Source: PLoS One. 2018 May 18;13(5):e0194368. doi: 10.1371/journal.pone.0194368 (PMC5959193; doi:10.1371/journal.pone.0194368)
Supplement: S4 Table — (DOCX) [file pone.0194368.s012.docx]

**Table S4**. **List of predicted restriction-modification systems in the 18 genomes of the genus *Pseudovibrio****.*

| **Locus tag** | **R-M classification** |
| --- | --- |
| **Organism**: *Pseudovibrio* sp. FO-BEG1 | |
| FOBEG_00264 | Solitary REase |
| FOBEG_00736  FOBEG_00737  FOBEG_00739 | Type I R-M |
| FOBEG_01576 | Solitary MTase |
| FOBEG_03980 | Solitary REase |
| FOBEG_04360 | Solitary REase |
| **Organism**: *Pseudovibrio* sp. JE062 | |
| JE_062_01971 | Solitary REase |
| JE_062_02647 | Solitary MTase |
| JE_062_03183  JE_062_03185 | Type II R-M |
| JE_062_03482  JE_062_03483  JE_062_03486 | Type I R-M |
| JE_062_03840 | Solitary REase |
| JE_062_04190 | Solitary REase |
| JE_062_04257  JE_062_04258  JE_062_04259 | Type I R-M |
| JE_062_04262 | Solitary REase |
| **Organism**: *Pseudovibrio* sp. POLY-S9 | |
| PPL9_01789 | Solitary REase |
| PPL9_01606 | Solitary MTase |
| PPL9_02004 | Solitary REase |
| PPL9_02398 | Solitary MTase |
| PPL9_02817 | Solitary MTase |
| PPL9_03090 | Solitary REase |
| PPL9_03689 | Solitary MTase |
| PPL9_04035 | Solitary MTase |
| PPL9_04039 | Solitary MTase |
| PPL9_04810 | Solitary MTase |
| PPL9_05489 | Solitary MTase |
| PPL9_06035 | Solitary MTase |
| PPL9_05516 | Solitary MTase |
| **Organism**: *Pseudovibrio axinellae* AD2 | |
| AD2_00388 | Solitary REase |
| AD2_01363 | Solitary MTase |
| AD2_02576 | Solitary MTase |
| AD2_03078 | Solitary MTase |
| AD2_03092 | Solitary MTase |
| AD2_03266 | Solitary MTase |
| AD2_03344  AD2_03346  AD2_03347 | Type I R-M |
| AD2_03409 | Solitary REase |
| AD2_03722 | Solitary REase |
| **Organism**: *Pseudovibrio* sp. AD13 | |
| AD13_01409 | Solitary REase |
| AD13_01430 | Solitary MTase |
| AD13_01730 | Solitary MTase |
| AD13_03235 | Solitary MTase |
| AD13_03236 | Solitary MTase |
| AD13_03701 | Solitary MTase |
| AD13_04257 | Solitary MTase |
| AD13_04331 | Solitary MTase |
| AD13_05037 | Solitary MTase |
| **Organism**: *Pseudovibrio* sp. AD14 | |
| AD14_01466 | Solitary MTase |
| AD14_01836 | Solitary REase |
| AD14_02595 | Solitary MTase |
| AD14_03389 | Solitary MTase |
| AD14_03975 | Solitary MTase |
| AD14_03989 | Solitary MTase |
| AD14_04026 | Solitary MTase |
| AD14_04566 | Solitary REase |
| AD14_04940 | Solitary REase |
| AD14_05737 | Solitary MTase |
| Organism: *Pseudovibrio* sp. AD26 | |
| AD26_01502 | Solitary MTase |
| AD26_01757 | Solitary MTase |
| AD26_03813  AD26_03814  AD26_03815 | Type II R-M |
| AD26_03857 | Solitary MTase |
| AD26_04199  AD26_04200  AD26_04201 | Type I R-M |
| AD26_04282 | Solitary REase |
| AD26_04661 | Solitary MTase |
| AD26_04750 | Solitary REase |
| AD26_05481 | Solitary MTase |
| AD26_05526 | Solitary MTase |
| AD26_05666  AD26_05668  AD26_05669 | Type I R-M |
| AD26_05720  AD26_05721  AD26_05722 | Type I R-M |
| AD26_05726 | Solitary REase |
| **Organism**: *Pseudovibrio* sp. AD37 | |
| AD37_00257 | Solitary REase |
| AD37_00786 | Solitary REase |
| AD37_00789  AD37_00790  AD37_00791 | Type I R-M |
| AD37_03704 | Solitary REase |
| AD37_04074 | Solitary REase |
| AD37_05357  AD37_05359  AD37_05360 | Type I R-M |
| **Organism**: *Pseudovibrio* sp. AD46 | |
| AD46_01546 | Solitary MTase |
| AD46_03379  AD46_03380 | Type II R-M |
| AD46_03793 | Solitary MTase |
| AD46_03880 | Solitary MTase |
| AD46_04011 | Solitary REase |
| AD46_04324  AD46_04326  AD46_04327 | Type I R-M |
| AD46_04397 | Solitary REase |
| AD46_05222 | Solitary REase |
| **Organism**: *Pseudovibrio* sp. AD5 | |
| AD5_01417 | Solitary MTase |
| AD5_02744 | Solitary MTase |
| AD5_03601 | Solitary MTase |
| AD5_03686  AD5_03687 | Type II R-M |
| AD5_03714 | Solitary MTase |
| AD5_03726 | Solitary MTase |
| AD5_03836 | Solitary MTase |
| AD5_03840 | Solitary MTase |
| AD5_04217 | Solitary REase |
| AD5_04654 | Solitary REase |
| AD5_05383 | Solitary MTase |
| AD5_05392 | Solitary MTase |
| AD5_05419  AD5_05420 | Type II R-M |
| AD5_05423 | Solitary MTase |
| AD5_05521 | Solitary REase |
| **Organism**: *Pseudovibrio* sp. W64 | |
| W64_01662 | Solitary MTase |
| W64_03925 | Solitary MTase |
| W64_04406 | Solitary REase |
| W64_04409  W64_04410  W64_04411 | Type I R-M |
| W64_04606 | Solitary REase |
| W64_04955 | Solitary REase |
| **Organism**: *Pseudovibrio* sp. W74 | |
| W74_01041 | Solitary MTase |
| W74_01055 | Solitary MTase |
| W74_01989 | Solitary MTase |
| W74_02413 | Solitary REase |
| W74_03979 | Solitary MTase |
| W74_04511 | Solitary MTase |
| W74_04127 | Solitary MTase |
| W74_04782 | Solitary REase |
| W74_05153 | Solitary REase |
| W74_05676 | Solitary MTase |
| **Organism**: *Pseudovibrio* sp. WM33 | |
| WM33_00258 | Solitary REase |
| WM33_01086 | Solitary REase |
| WM33_01210 | Solitary MTase |
| WM33_01810 | Solitary MTase |
| WM33_02038 | Solitary MTase |
| WM33_03313 | Solitary REase |
| WM33_04231 | Solitary MTase |
| WM33_04268 | Solitary MTase |
| WM33_04740 | Solitary REase |
| WM33_05196 | Solitary MTase |
| WM33_05413  WM33_05415 | Type II R-M |
| **Organism**: *P. ascidiaceicola* DSM-16392 | |
| PAS_16_01373 | Solitary MTase |
| PAS_16_01623 | Solitary MTase |
| PAS_16_01921  PAS_16_03352 | Solitary REase  Solitary REase |
| PAS_16_04255 | Solitary MTase |
| PAS_16_04339 | Solitary REase |
| PAS_16_04693 | Solitary REase |
| **Organism**: *Pseudovibrio* sp. Tun.PHSC04_5.I4 | |
| TUN_51_00110 | Solitary MTase |
| TUN_51_01120 | Solitary MTase |
| TUN_51_01121 | Solitary REase |
| TUN_51_01234 | Solitary MTase |
| TUN_51_01651 | Solitary MTase |
| TUN_51_01671  TUN_51_01677  TUN_51_01680  TUN_51_01681  TUN_51_01682 | Type I R-M |
| TUN_51_01897 | Solitary REase |
| TUN_51_02226 | Solitary MTase |
| TUN_51_02235 | Solitary MTase |
| TUN_51_02272 | Solitary REase |
| TUN_51_03698 | Solitary MTase |
| TUN_51_03719 | Solitary MTase |
| TUN_51_03991 | Solitary MTase |
| TUN_51_04211 | Solitary MTase |
| TUN_51_04220 | Solitary MTase |
| TUN_51_04266 | Solitary MTase |
| TUN_51_04673  TUN_51_04674  TUN_51_04675 | Type I R-M |
| TUN_51_04669 | Solitary REase |
| TUN_51_04681 | Solitary MTase |
| TUN_51_04693 | Solitary MTase |
| TUN_51_04833 | Solitary MTase |
| TUN_51_04834  TUN_51_05579 | Solitary MTase  Solitary MTase |
| TUN_51_05994 | Solitary MTase |
| TUN_51_06214 | Solitary MTase |
| TUN_51_06215 | Solitary MTase |
| TUN_51_06126  TUN_51_06127 | Type II R-M |
| TUN_51_06132 | Solitary MTase |
| TUN_51_06133 | Solitary REase |
| **Organism**: *P. stylochi* UST20140214-052 | |
| P052_00008 | Solitary REase |
| P052_01452 | Solitary MTase |
| P052_01515 | Solitary REase |
| P052_02627 | Solitary REase |
| P052_02943 | Solitary REase |
| P052_03144 | Solitary MTase |
| **Organism**: *P. hongkongensis* UST20140214-015B | |
| P015B_01111 | Solitary MTase |
| P015B_01112 | Solitary MTase |
| P015B_01351 | Solitary MTase |
| P015B_02212 | Solitary MTase |
| P015B_02858 | Solitary REase |
| P015B_03082 | Solitary MTase |
| P015B_03197 | Solitary REase |
| P015B_03488 | Solitary REase |
| **Organism**: *P. denitrificans* JCM12308 | |
| JCM123_01716 | Solitary MTase |
| JCM123_04502 | Solitary REase |
| JCM123_04898 | Solitary REase |
| JCM123_05448  JCM123_05449 | Type I R-M |
