# Supplementary material for: Genus-wide comparison of Pseudovibrio bacterial genomes reveal diverse adaptations to different marine invertebrate hosts
Source: PLoS One. 2018 May 18;13(5):e0194368. doi: 10.1371/journal.pone.0194368 (PMC5959193; doi:10.1371/journal.pone.0194368)
Supplement: S5 Table — P-values are based on t-test. (DOCX) [file pone.0194368.s013.docx]

**Table S5.** Comparison of proportion of functional genes encoded in the *Pseudovibrio* genomes and their genomic islands. *P-*values are based on t-test.

|  | Average percentage (%) | | Genome-GI |
| --- | --- | --- | --- |
| COG categories | Genome | Genomic Island (GI) | *p*-value |
| B: chromatin structure and dynamics | 0.0700 | 0 | **** |
| C: energy production and conversion | 5.755 | 2.495 | **** |
| D: cell cycle control, cell division, chromosome partitioning | 0.5888 | 1.331 | ** |
| E: amino acid transport and metabolism | 10.56 | 4.6100 | **** |
| F: nucleotide transport and metabolism | 2.126 | 0.9500 | *** |
| G: carbohydrate transport and metabolism | 7.41 | 4.1300 | *** |
| H: coenzyme transport and metabolism | 3.972 | 2.6700 | ** |
| I: lipid transport and metabolism | 4.002 | 3.9900 | - |
| J: translation, ribosomal structure and biogenesis | 4.505 | 2.495 | ** |
| K: transcription | 8.657 | 11.2700 | ** |
| L: replication, recombination and repair | 3.623 | 16.4400 | **** |
| M: cell wall/membrane/envelope biogenesis | 4.722 | 7.7000 | ** |
| N: cell motility | 2.185 | 0.5800 | **** |
| O: posttranslational modification, protein turnover, chaperones | 3.461 | 2.8700 | - |
| P: inorganic ion transport and metabolism | 5.248 | 2.5600 | *** |
| Q: secondary metabolite biosynthesis, transport and catabolism | 3.119 | 5.7500 | ** |
| R: general function prediction only | 13.37 | 14.2800 | - |
| S: function unknown | 9.174 | 9.4300 | - |
| T: signal transduction mechanisms | 4.146 | 1.6300 | **** |
| U: intracellular trafficking, secretion, and vesicular transport | 1.97 | 1.2400 | * |
| V: defense mechanisms | 1.29 | 3.5900 | ** |
| W: extracellular structure | 0 | 0 | - |
| Z: cytoskeleton | 0 | 0 | - |

**p*<0.05, ***p*<0.005, ****p*<0.001, *****p*<0.00001
