# Supplementary material for: Genus-wide comparison of Pseudovibrio bacterial genomes reveal diverse adaptations to different marine invertebrate hosts
Source: PLoS One. 2018 May 18;13(5):e0194368. doi: 10.1371/journal.pone.0194368 (PMC5959193; doi:10.1371/journal.pone.0194368)
Supplement: S6 Table — P-values are basedon t-test. (DOCX) [file pone.0194368.s014.docx]

**Table S6**. Comparison of proportion of genes encoded for subcellular localization in the *Pseudovibrio* genomes and their genomic islands. *P-*values are based on t-test.

|  | Average percentage (%) | | Genome-GI |
| --- | --- | --- | --- |
| **Subcellular location** | **Genome** | **Genomic Island (GI)** | ***p*-value** |
| Cytoplasmic | 43.88 | 37.51 | *** |
| CytoplasmicMembrane | 21.89 | 12.11 | **** |
| Extracellular | 1.26 | 2.20 | * |
| OuterMembrane | 1.13 | 0.44 | **** |
| Periplasmic | 3.36 | 0.69 | **** |
| Unknown | 28.3 | 47.04 | **** |

**p*<0.05, ****p*<0.001, *****p*<0.00001
